# Human-specific elimination of epithelial Siglec-XII suppresses the risk of inflammation-driven colorectal cancers

**DOI:** 10.1172/jci.insight.181539

**Published:** 2024-07-11

**Authors:** Hector A. Cuello, Saptarshi Sinha, Andrea L. Verhagen, Nissi Varki, Ajit Varki, Pradipta Ghosh

**Affiliations:** 1 Department of Cellular & Molecular Medicine,; 2 Glycobiology Research and Training Center,; 3 Department of Pathology,; 4 Department of Medicine,; 5 Center for Academic Research and Training in Anthropogeny,; 6 Moores Comprehensive Cancer Center, and; 7 HUMANOID Center of Research Excellence (CoRE), University of California, San Diego, La Jolla, California, USA.

**Keywords:** Inflammation, Oncology, Cancer, Colorectal cancer

## Abstract

Carcinomas are common in humans but rare among closely related “great apes.” Plausible explanations, including human-specific genomic alterations affecting the biology of sialic acids, are proposed, but causality remains unproven. Here, an integrated evolutionary genetics-phenome-transcriptome approach studied the role of *SIGLEC12* gene (encoding Siglec-XII) in epithelial transformation and cancer. Exogenous expression of the protein in cell lines and genetically engineered mice recapitulated approximately 30% of the human population in whom the protein is expressed in a form that cannot bind ligand because of a fixed, homozygous, human-universal missense mutation. Siglec-XII–null cells/mice recapitulated the remaining approximately 70% of the human population in whom an additional polymorphic frameshift mutation eliminates the entire protein. Siglec-XII expression drove several pro-oncogenic phenotypes in cell lines and increased tumor burden in mice challenged with chemical carcinogen and inflammation. Transcriptomic studies yielded a 29-gene signature of Siglec-XII–positive disease and when used as a computational tool for navigating human data sets, pinpointed with surprising precision that *SIGLEC12* expression (model) recapitulates a very specific type of colorectal carcinomas (disease) that is associated with mismatch-repair defects and inflammation, disproportionately affects European Americans, and carries a favorable prognosis. They revealed a hitherto-unknown evolutionary genetic mechanism for an ethnic/environmental predisposition of carcinogenesis.

## Abstract 2

Our research on SIGLEC12’s impact on colorectal cancer, using an integrated evolutionary genetics-phenome-transcriptome approach, surprisingly revealed a novel genetic mechanism for ethnic environmental carcinogenesis.

## Graphical abstract

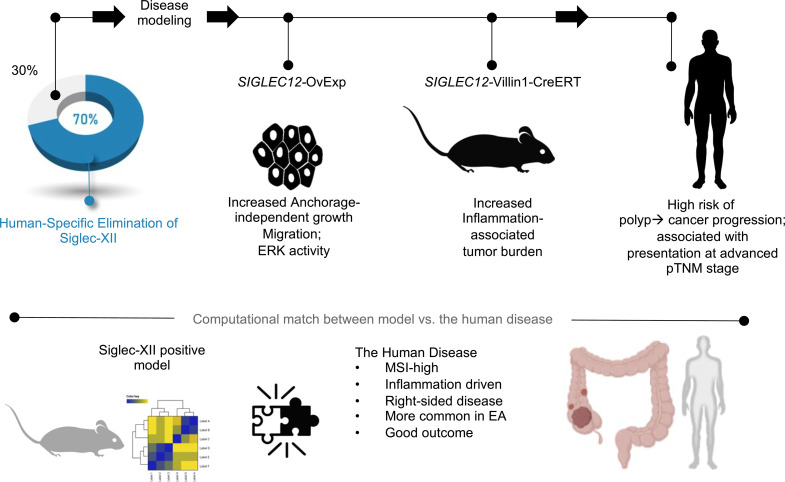

## Introduction

Colorectal cancers (CRCs) are the third most commonly diagnosed cancers and the second leading cause of cancer-related deaths globally, with an alarmingly rising incidence (1). Such high prevalence and rising incidence among humans are particularly surprising, given that CRCs among other many carcinomas are a rarity among captive chimpanzees, with whom we share more than 99% protein sequence homology (2–4). In fact, cancers are part of a list of common human diseases that may be partially or completely unique to our species compared with other primates (5–7).

Human-specific changes in specific Siglecs are proposed molecular mechanisms that could explain human proneness to developing cancers (8, 9). Siglecs are a group of vertebrate lectins belonging to the immunoglobulin superfamily that recognize glycan-bearing sialic acids (Sias). A subset of inhibitory CD33-related Siglecs (CD33rSiglecs) are prominently expressed in immune cells and are considered to have a regulatory role in suppressing the activation of innate immune cells via cytosolic immunoreceptor tyrosine-based inhibitory motifs (ITIMs) (10, 11). These ITIMs recruit protein phosphatases such as Src homology region 2-containing protein tyrosine phosphatases (PTPs) SHP1 and SHP2 (12, 13). Of relevance in the context of CRCs, both SHP1 (14) and SHP2 (15) have been found to serve as brakes that limit tumorigenesis via their ability to antagonistically inactivate pro-oncogenic tyrosine-based signals; mice lacking SHP1/2 in intestinal epithelial cells (IECs) develop higher tumor burden, associated with sustained activation of downstream pathways such as the PI3K/Akt, Wnt/β-catenin, NF-κB, and STAT3 signals. Thus, signaling via a functional Siglec→SHP1/2 axis in IECs is expected to inhibit tumorigenesis. Although multiple CD33rSiglecs are reported to be upregulated in cancers (16, 17), no study has evaluated the role of Siglecs in IECs.

Here we explore a previously unforeseen, human-specific association between CRCs and Siglec-XII, a member of the Siglec family of Sia-recognizing receptors that is primarily expressed in epithelial cells (18) and functionally inactivated from recognizing Sia ligands, thereby signaling aberrantly only in humans. Since mice do not have a *SIGLEC12* gene, we modeled the human disease in vitro and in mice and then used an unbiased computational approach to navigate human disease samples to unravel the implications of Siglec-XII expression and its impact on oncogenesis. Findings reveal that expression of a functionally defective Siglec-XII in a subset of humans predisposes them to develop a specific type of CRCs that is environmentally influenced (higher inflammation) and ethnically predisposed.

## Results

### A study design rationalized by human-specific evolutionary genetics of the SIGLEC12 gene.

**Figure 1:**
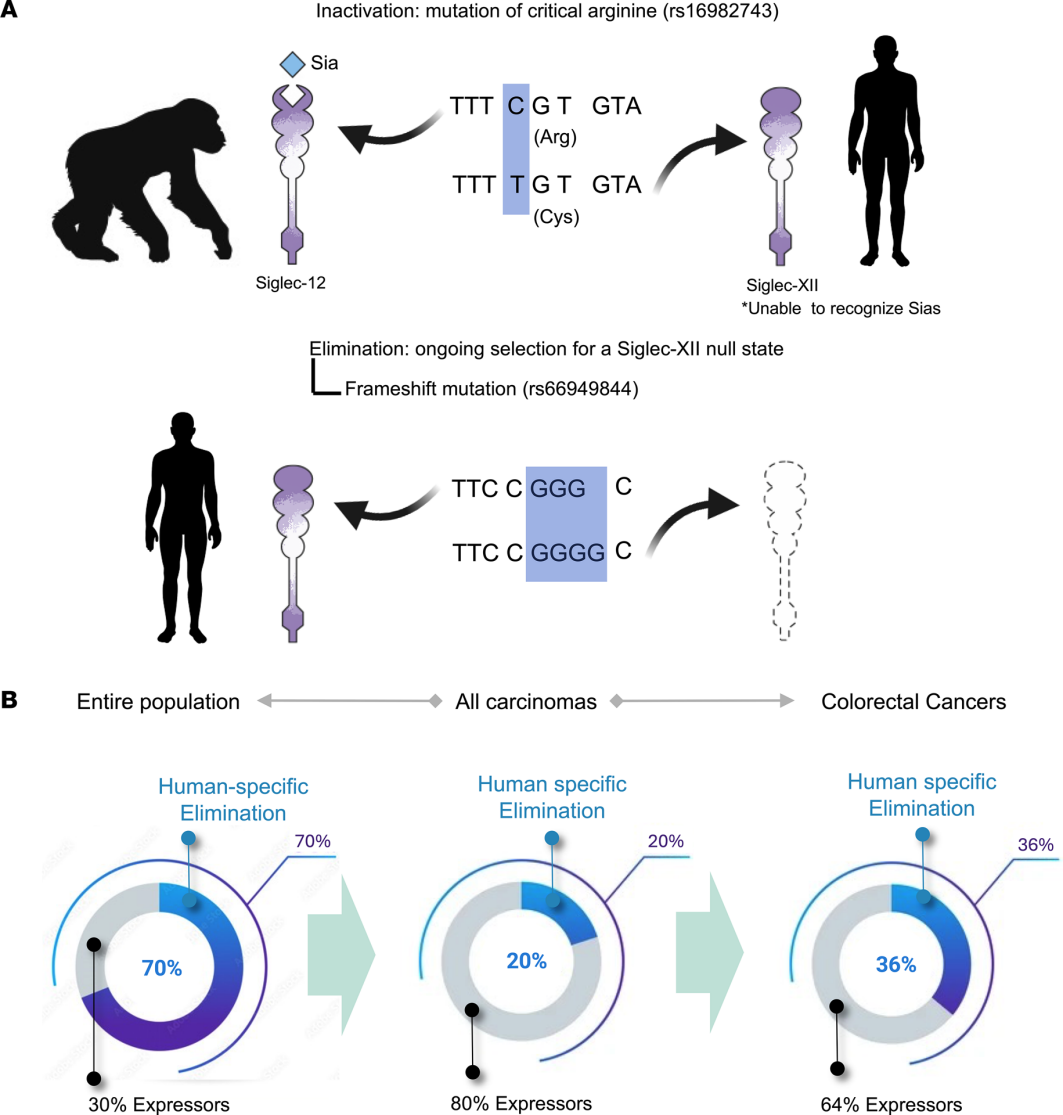
Mechanism and observed prevalence of human-specific inactivation and elimination of the protein product of *SIGLEC12*. (**A**) Schematic (top) summarizes the impact of the human-universal mutation (rs16982743) of the gene *SIGLEC12*, which results in a loss of an essential arginine that abolishes the ability of the Siglec-XII protein to bind/recognize sialic acids (Sias). This functionally inactivating mutation occurred prior to the common ancestor of all modern humans. *SIGLEC12* is intact and functional in great apes. Schematic (bottom) summarizes the ongoing selection for the Siglec-XII–null state that continues in the current worldwide human population. The most common polymorphic mutation is a frameshift mutation (rs66949844), guanine (G) insertion, which in the homozygous state eliminates the protein expression in most humans. (**B**) Pie charts indicate the restricted prevalence of Siglec-XII expression (~30%) in the entire human population (left) but the enrichment of such expressors among all (middle) and only colorectal (right) carcinomas.

To study the role of Siglec-XII in IECs and ensure that findings are relevant to the human disease (i.e., CRCs), we drew inspiration from the known uniquely human features of *SIGLEC12*, the gene that encodes Siglec-XII in humans. This gene harbors a human-universal missense mutation in the Sia-binding domain that affects a critical arginine residue (the Arg/Cys mutation), resulting in an inability to recognize Sias (hence the protein is denoted using Roman numeral XII to differentiate it from functional Siglecs) (19) (Figure 1A). This inactivating mutation occurred prior to the common ancestor of all modern humans and thus is absent in closely related “great apes”; the latter express a functional Siglec-12 preferentially recognizing Neu5Gc (a Sia lost from the human lineage) (18, 19). Furthermore, the human *SIGLEC12* locus is currently undergoing negative selection in humans that favors a null and/or truncated form of the protein, characterized by the excess of rare alleles and the presence of “selective sweep” acting on the gene throughout the overall human population (20). The most common frameshift mutation arises from the insertion of a guanine (G) in the *SIGLEC12* gene, resulting in the loss of expression of the complete protein in most humans (Figure 1A) (18, 21). Interestingly, among the minority of humans who possess the genomic ability to express it, the protein is detected in certain tissue macrophages but is not found in other blood cell types and instead exhibits higher levels of expression on the surfaces of epithelial cells (18). Another peculiarity of this protein is that, even though Siglec-XII does not have the ability to recognize Sias, it still possesses ITIM and ITIM-like domains in its cytosolic tail that can undergo phosphorylation to recruit Shp1 and Shp2 phosphatases (22), raising the possibility of Siglec-XII serving as a dominant-negative protein that can signal (via Shp) in the absence of binding the natural ligand (Sias).

We hypothesized that aberrant signaling via Siglec-XII supports human-specific mechanisms increasing cancer risk and progression. Because the ligand binding–defective mutant is expressed in only approximately 30% of the healthy human population but enriched up to ~70%–80% in all carcinomas and ~64% in CRCs (8) (Figure 1B), we hypothesized that the minority of humans who express full-length Siglec-XII may be at the highest risk for developing advanced carcinomas. The enrichment of Siglec-XII positivity from normal to cancer tissues suggested that Siglec-XII positivity, either alone or via its interaction with environmental factors, predisposes to cancers. To model these uniquely human features of the *SIGLEC12* gene, we exogenously expressed the ligand-binding defective Siglec-XII in cell lines and in mice (which do not have a *SIGLEC12* gene, ref. 13) and explored cellular phenotypes and tumorigenesis, respectively.

### Exogenous expression of Siglec-XII in null human carcinoma cell lines enhances malignant features.

Previously, a flow cytometry screen of 5 human carcinoma cell lines showed that MDA-PCa-2b and LNCaP (prostate cancer) and MCF-7 (breast cancer) lines express Siglec-XII, whereas MDA-MB-231 (breast cancer) and PC-3 (prostate cancer) do not express it (18). In a second screen of another set of 4 CRC cell lines (Colo-320, Caco-2, LS-180, and HT-29), also by flow cytometry, we verified that 2 of 4 expressed the Siglec-XII protein (LS-180 and HT-29) (Supplemental Figure 1A; supplemental material available online with this article; https://doi.org/10.1172/jci.insight.181539DS1). We checked for and confirmed the absence of the polymorphic frameshift insertion mutation in the human *SIGLEC12* gene (Supplemental Figure 1B), the event which results in a premature stop codon and consequent loss of expression in most individuals. The higher frequency of expression in carcinoma cell lines (5/9) is in keeping with the prior conclusion (8) that human carcinomas have a higher incidence of Siglec-XII expression than expected in the general population.

**Figure 2:**
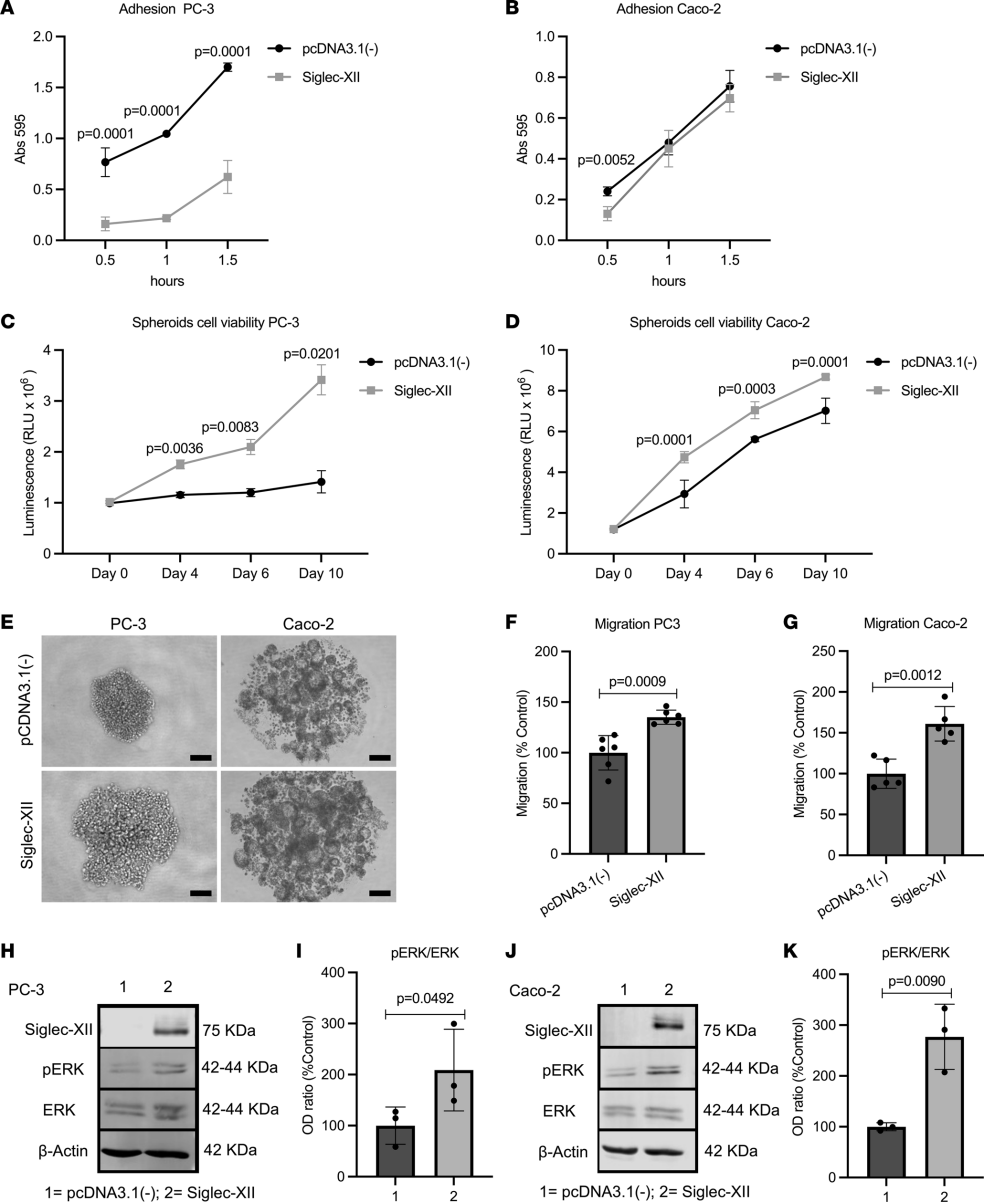
Forced expression of Siglec-XII in null human carcinoma cell lines enhances cellular processes associated with tumor aggressiveness. (**A** and **B**) Graphs display cell adhesion on a 2D surface for PC-3 (**A**) and Caco-2 (**B**) cells, as measured by crystal violet staining. (**C**–**E**) Graphs (**C**, PC-3; **D**, Caco-2) display cellular viability of the same cells in 3D tumoroid cultures. Representative images are displayed (**E**). Scale bar: 100 μm. (**F** and **G**) Graphs display percentage migration of PC-3 (**F**) and Caco-2 (**G**) cells, as determined by Transwell assays (0%–10% serum gradient). (**H**–**K**) Quantitative immunoblotting on equal aliquots of whole cell lysates of PC-3 (**H** and **I**) or Caco-2 (**J** and **K**) cells to assess ERK1/2 activity. Blots results were set up in parallel, run contemporaneously, and normalized to loading controls (β-Actin). OD, optical density. Representative immunoblots are shown in **H** and **J**, and quantification of 3 independent repeats is shown as bar graphs in **I** and **K**. Error bars indicate ± SD. See also Supplemental Figure 1 for approaches used to verify the Siglec-XII–null state. Statistics: *P* values were calculated using GraphPad Prism. *P* < 0.05 was considered significant. **A**–**D**, ANOVA followed by Tukey’s multiple comparisons post hoc test. **F**, **G**, and **K**, 2-tailed *t* test. **I**, 1-tailed *t* test.

To begin to explore the significance of Siglec-XII in the progression of CRCs, we used Siglec-XII–nonexpressing Caco-2 cells. As positive control, and to provide continuity with our prior work (8, 18), we used Siglec-XII–nonexpressing PC-3 human prostate carcinoma cells. These cells were previously characterized by flow cytometry to confirm cell surface expression of Siglec-XII exclusively after transfection. Both cell lines were transfected with either pCDNA-3.1-*SIGLEC12* or with empty vector (control), forcing them to exogenously express full-length Siglec-XII. Stable clones were selected (see Methods) and used in a variety of assays, i.e., cell adhesion, spheroid formation, and migration. Compared with controls, the Siglec-XII–expressing counterparts significantly and consistently showed decreased cell adhesion (Figure 2, A and B), accelerated spheroid growth (Figure 2, C–E), and increased Transwell migration (Figure 2, F and G). These phenotypes were accompanied also by increased ERK1/2 activity, as determined by immunoblotting for the phosphorylated kinase (Figure 2, H–K). Siglec-XII expression was verified by immunoblotting (Figure 2, H–K).

### Creation and validation of a transgenic intestine-specific knockin SIGLEC12 murine model.

**Figure 3:**
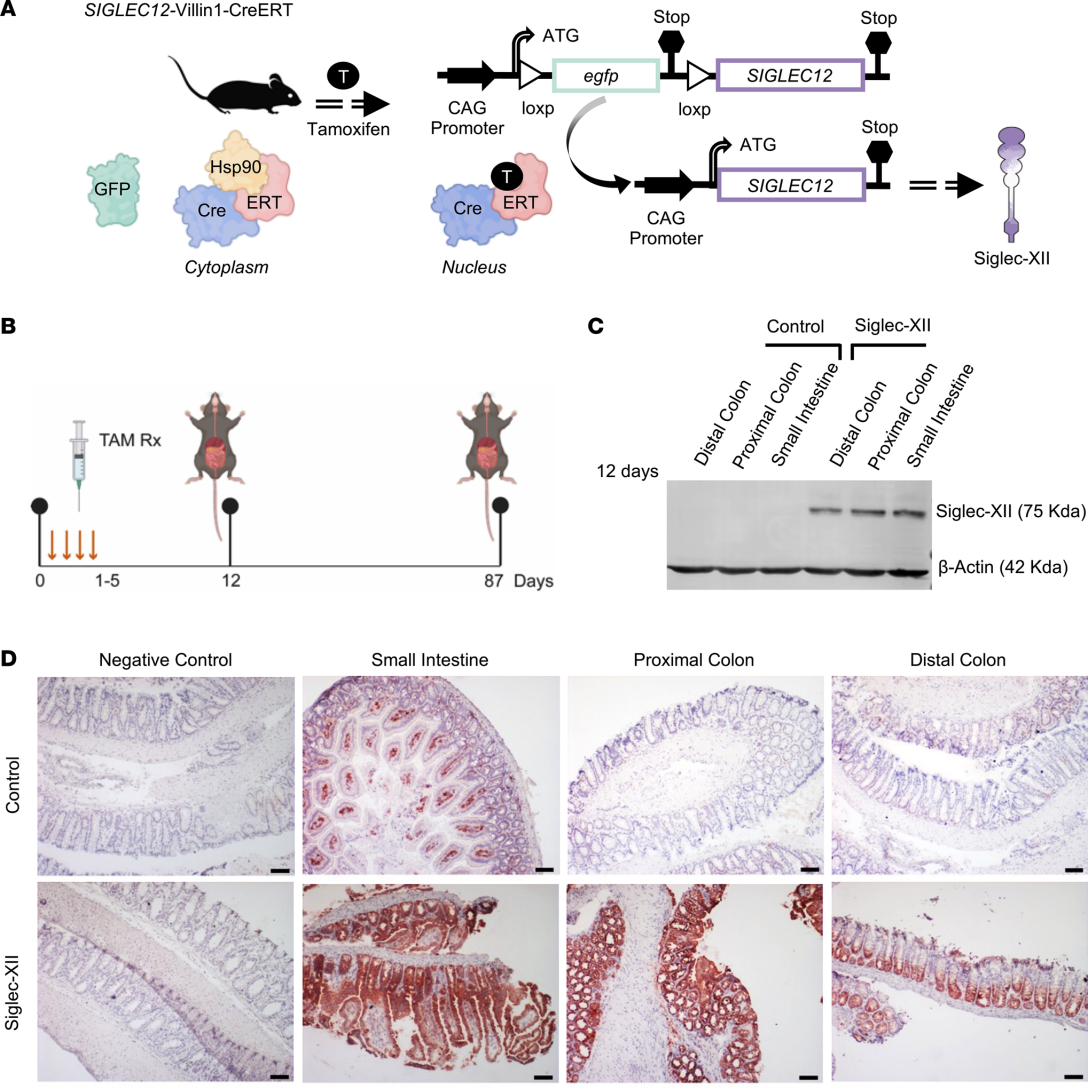
Creation and validation of a transgenic knockin *SIGLEC12* murine model that expresses Siglec-XII in the small and large intestine. (**A**) Schematic displays the cloning strategy for creation of the *SIGLEC12*–knockin mice (wild-type mice do not harbor a *SIGLEC12* gene). Tissue-specific Cre-driver mice express a tamoxifen-inducible system of estrogen receptor fused to Cre (Cre-ERT). In the absence of tamoxifen (T), Hsp90 binds to Cre-ERT and maintains its cytoplasmic retention. Nuclear translocation of Cre-ERT by tamoxifen. In the nucleus, Cre-ERT recognizes *loxP* sites and allows tissue-specific expression of Siglec-XII. (**B**) An overview of experimental design for the induction of Siglec-XII expression by serial administration of tamoxifen on 5 consecutive days, followed by harvesting of tissues to confirm early (day 12) and sustained (day 87) expression of Siglec-XII. (**C**) Western blot for Siglec-XII and β-actin on transgenic mouse and control tissues at day 12 after induction using tamoxifen. (**D**) Expression of Siglec-XII in mouse tissue evaluated by immunohistochemistry at day 12 after induction using tamoxifen. Scale bar: 100 μm. See also Supplemental Figure 2A (for immunoblots) and Supplemental Figure 2B (for immunohistochemistry) on samples at day 87 after induction.

Because carcinogenesis in the colon requires complex interplay between multiple factors (host genetics, gut microbes, and the immune system) that are hard to recapitulate in vitro in cell line models, mouse models have proven crucial in the identification of the role of genes responsible for CRC initiation and progression (23). Given that mice do not have any endogenous *SIGLEC12* gene (13), we developed a mouse model that allows conditional expression of the protein Siglec-XII (see legend, Figure 3A). The *SIGLEC12*-knockin mice present the *egfp* gene (including stop codon) flanked between 2 *loxP* sites, and it is upstream of the Siglec-XII coding sequence. This stop codon prevents *SIGLEC12* gene expression in the absence of Cre recombinase. To selectively knock in *SIGLEC12* in the intestine, we bred *SIGLEC12*-transgenic mice with Villin1-Cre-ERT mice; the latter restricts the Cre-ERT expression to the villi and crypts of the small and large intestine (24, 25). We collected small and large intestines at early and late points after 5 consecutive days of tamoxifen administration (Figure 3B) and analyzed them for Siglec-XII expression by immunohistochemistry and immunoblotting. Both methodologies verified that Siglec-XII is expressed prominently and homogeneously exclusively in the transgenic mice (*SIGLEC12*-Villin1-Cre-ERT) but not their control littermates (Villin1-Cre-ERT) as early as day 12 (Figure 3, C and D). We also verified that such conditional expression of Siglec-XII in the transgenic mice was sustained as late as day 87 (Supplemental Figure 2). Histopathological analysis ruled out any gross or microscopic changes on day 87 in various organs (colon, liver, kidney, lung, and spleen) (Supplemental Figure 3). More importantly, we did not observe any features suggestive of inflammation, metaplasia, dysplasia, or neoplasia.

### SIGLEC12-knockin mice display greater tumor burden in response to chemical carcinogenesis.

**Figure 4:**
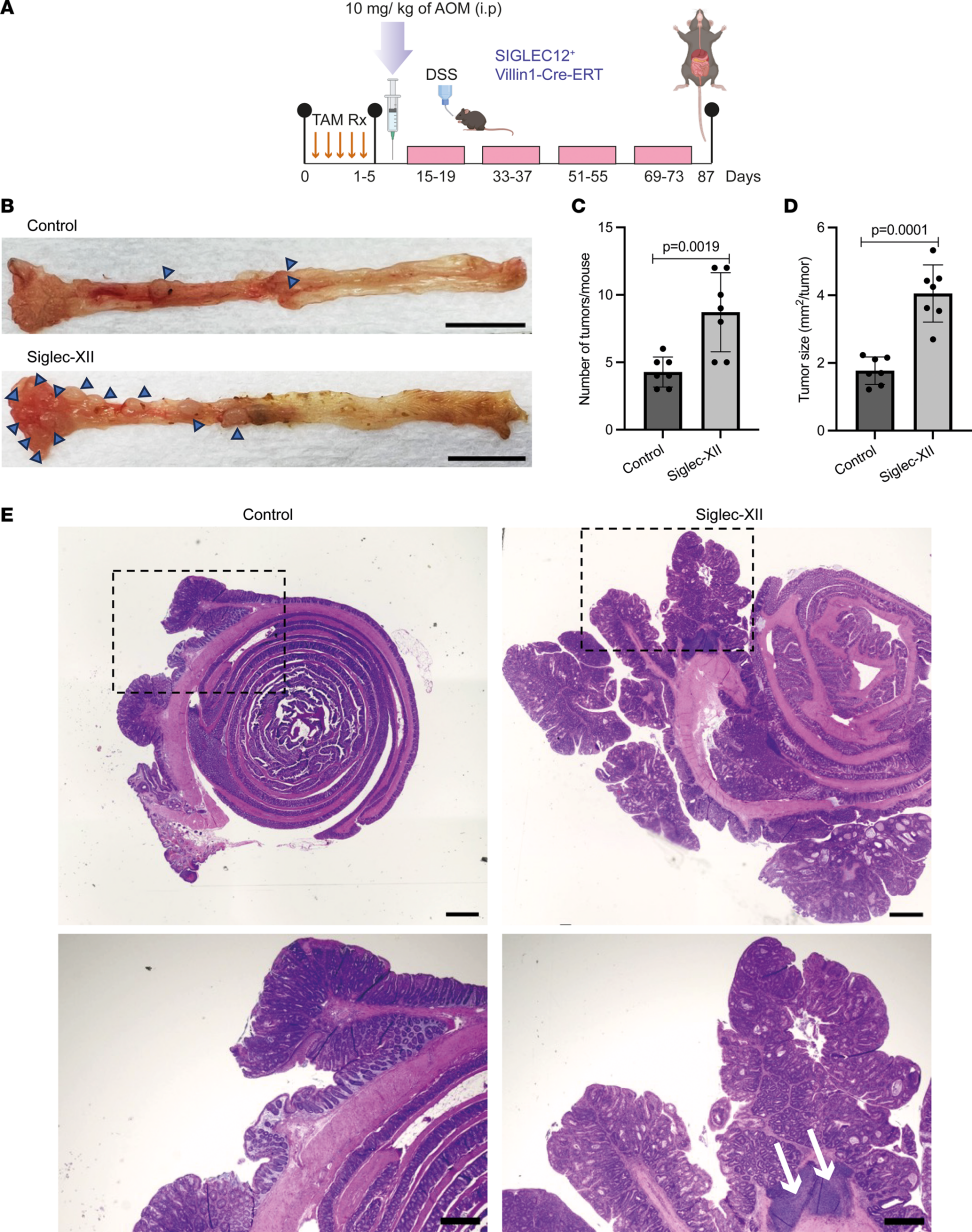
Transgenic knockin *SIGLEC12* mice are at greater risk of inflammation-associated CRCs. (**A**) An overview of experimental design for the induction of Siglec-XII expression by serial administration of tamoxifen followed by carcinogenesis protocol consisting of a single administration of azoxymethane (AOM) and 4 cycles of dextran sodium sulfate (DSS). (**B**) Representative pictures of colonic tissue from control and Siglec-XII–expressing mice subjected to tamoxifen administration and carcinogenesis protocol (AOM/DSS). Scale bar: 1 cm. The complete panel of pictures of colonic tissue is shown in Supplemental Figure 4. (**C** and **D**) Comparison of the number of tumors (**C**) and tumor size (**D**) in control (*N* = 7) and Siglec-XII–expressing mice (*N* = 7) subjected to tamoxifen administration and carcinogenesis protocol. Error bars indicate ± SD. (**E**) Representative pictures of H&E-stained colonic tissue from control and Siglec-XII–expressing mice subjected to tamoxifen administration and carcinogenesis protocol. The boxed areas (top) are shown at higher magnification (bottom). Arrows indicate immune cell infiltrates. Scale bars: 100 μm (top), 50 μm (bottom). Statistics: *P* values were calculated by 2-tailed *t* test using GraphPad Prism. *P* value < 0.05 was considered significant.

Because the transgenic *SIGLEC12* mice do not develop spontaneous tumors, next we sought to use the model in conjunction with chemically induced CRC models that recapitulate the progression from aberrant crypt foci and adenoma to adenocarcinoma and are commonly used to study the effects of diet, inflammation, and gut microbiota (23). More specifically, we subjected mice to chemical carcinogenesis using well-established azoxymethane (AOM) and dextran sulfate sodium salt (DSS) (26–28) (Figure 4A and Supplemental Figure 4). While AOM mainly leads to the generation of adenomas, exposure to AOM/DSS is known to induce the formation of a complete process of colon oncogenesis, progressing from the initial proliferation of crypts to the final development of high-grade dysplasia and adenocarcinomas in ~25%–50% of C57BL/6 mice (29). Because many Siglecs are inhibitory receptors expressed in innate immune cells that regulate inflammation (30), the AOM/DSS model seemed furthermore appropriate as it is known to primarily recapitulate inflammation-driven CRCs (31). The animals were followed for 87 days and examined for colorectal tumors at necropsy. Examination of the colons showed that Siglec-XII–expressing mice presented significantly increased tumor burden over controls (Figure 4, B and C), and the bases of the tumors were typically associated with immune cell infiltrates (arrows, compare Figure 4, D and E). Animals with induced Siglec-XII and exposure to AOM/DSS also showed larger rectal tumors compared with control animals (Figure 4, D and E).

### Gene signatures uniquely induced because of Siglec-XII expression in human CRCs.

**Figure 5:**
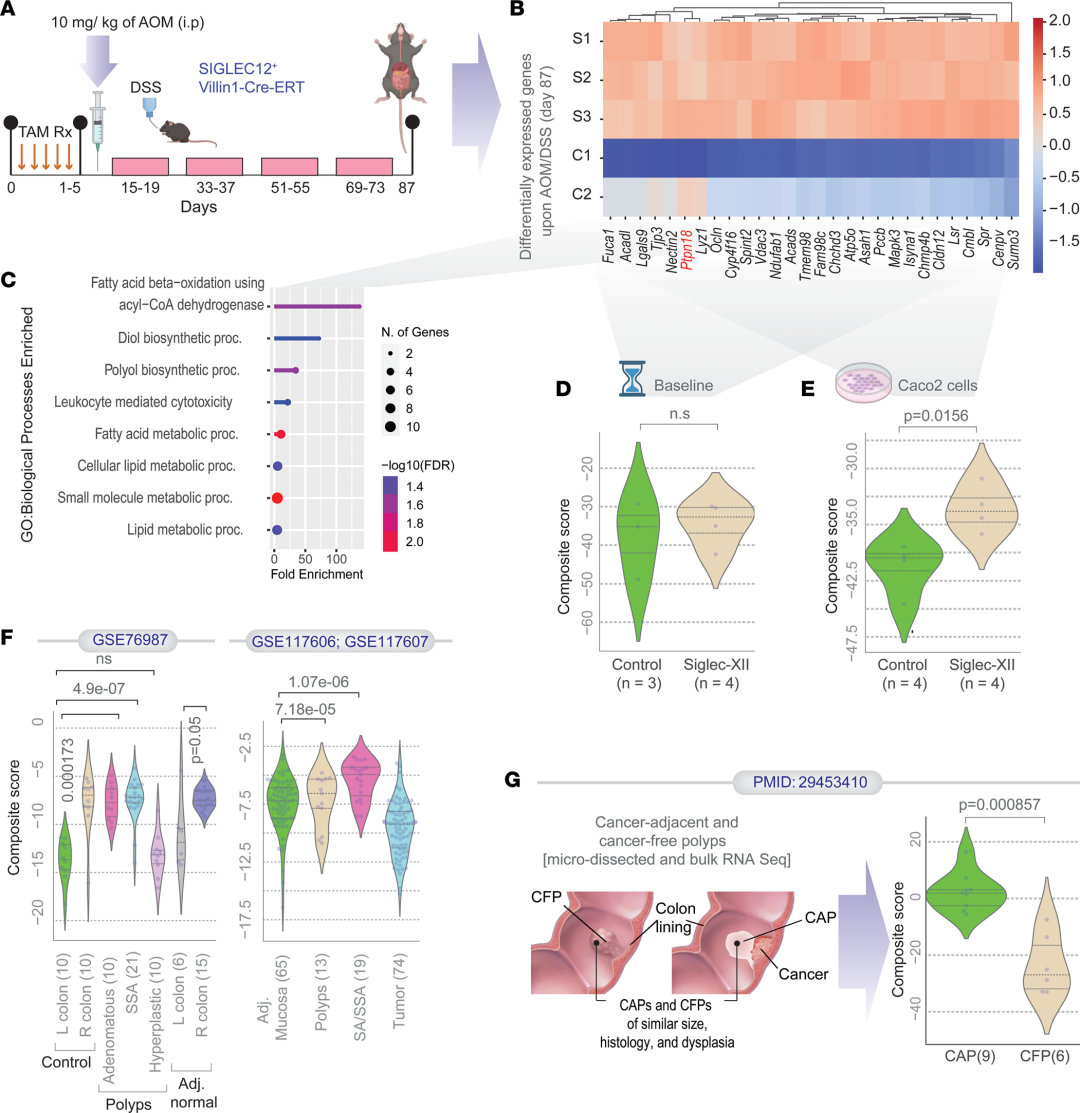
S*IGLEC12* expression induces gene expression in polyps at risk of progression to CRCs. (**A**) An overview of experimental design for the induction of Siglec-XII expression by serial administration of tamoxifen followed by carcinogenesis protocol consisting of a single administration of AOM and 4 cycles of DSS. On day 87, mouse colon tumors were harvested for RNA-Seq analysis. (**B**) Heatmap shows the *z*-normalized expression pattern of upregulated differentially expressed genes (DEGs) in Siglec-XII–expressing cohort. Red on the x axis highlights *Ptpn18* gene. (**C**) Plot showing the fold-enrichment of various biological processes from the Gene Ontology (GO) database. (**D** and **E**) Violin plots display the StepMiner normalized composite scores of the DEGs (in **B**) in control versus Siglec-XII samples at baseline (day 12; **D**) (collected within 1 week after tamoxifen administration) and Caco-2 pcDNA3.1(-) and Siglec-XII–Caco-2 (**E**). (**F**) Violin plots display the StepMiner normalized composite scores of the DEGs (in **B**) in patient tissues from 3 independent cohorts (National Center for Biotechnology Information [NCBI] Gene Expression Omnibus [GEO] GSE76987, GSE117606, and GSE117607). R, right; L, left; SSA, sessile serrated adenoma; Adj., adjacent. (**G**) Violin plots display the StepMiner normalized composite scores of the DEGs (in **B**) in laser-microdissected adenomatous tissues from polyps that progressed to cancers (cancer-adjacent polyps [CAPs]) versus those that did not (cancer-free polyps [CFPs]) (33–35). Statistics: *P* values in each violin plot (**D**–**G**) are based on 2-tailed Welch’s *t* test between comparator groups. *P* values for survival plots were determined by log-rank test.

To ascertain which processes or drivers of human carcinogenesis are recapitulated in our chemically induced transgenic mice, we sought an unbiased, computationally driven, 2-step approach. First, we carried out RNA-Seq of the colons at baseline and after AOM/DSS challenge. A differential expression analysis of genes between AOM/DSS-treated controls (Villin1-Cre-ERT) and Siglec-XII–expressing mice led to the identification of a 29-gene signature (Figure 5, A and B), which was upregulated in Siglec-XII mice. This set of 29 genes was enriched for diverse bioenergetic processes (Figure 5C). As expected, in the absence of ligand recognition capabilities, the tyrosine-based signaling pathways, typically modulated antagonistically by Shp1/2 phosphatases, were lacking. These DEGs were not differentially expressed at early time points (baseline; Figure 5D), when Siglec-XII expression was strong (Figure 3, C and D), indicating that Siglec-XII expression alone was insufficient. Instead, the gene signature captured the combinatorial effect of AOM/DSS and Siglec-XII. In fact, no significant DEGs were found between baseline samples. The DEGs were upregulated also in Siglec-XII–expressing Caco-2 cells (Figure 5E).

Next, we used the gene set as a signature of CRC predisposition to navigate diverse CRC data sets. Because chemical induction models recapitulate some of the earliest steps for CRC initiation and progression (23), such as aberrant crypt foci and dysplasia, we asked if the gene signature is differentially induced in different parts of the human colon and diverse subtypes of polyps that carry differential risk of progression to CRCs. We found that the 29-gene signature uniquely induced due to Siglec-XII was induced also in the right side of the human colon (compared with the left; see Figure 5F), regardless of whether these samples were from healthy participants (Control; Figure 5F) or from patients who had polyps (Adj. normal; Figure 5F). The signature was significantly induced in polyps that are known to carry risk for CRC progression (adenomatous and sessile serrated adenomas; SSAs; Figure 5F) but not in benign hyperplastic polyps. Induction of the signature in polyps was verified in an independent cohort (Figure 5F).

We asked if Siglec-XII expression is associated with higher risk of polyp-to-CRC progression. To this end, we leveraged a publicly available data set that represents a time-lapse model for CRC initiation and progression in humans (32) (Figure 5G). In that model, CAPs were used as a model to study cancer progression temporally because the precursor polyp of origin remains in direct contiguity with its related tumor (33–35). CFP cases, on the other hand, are polyps that have remained cancer free, despite sharing similar size, histologic features, and degrees of dysplasia as CAPs (Figure 5G). Laser-dissected preneoplastic tissues from the CAPs represent polyps with a proven high risk of CRCs, CFPs represent polyps at low risk, and paired normal colons sampled approximately 8 cm away from the polyps serve as controls. Our 29-gene signature was significantly induced in CAPs compared with CFPs and could classify them perfectly (receiver operating characteristic [ROC] AUC 1.00; Figure 5G), indicating that Siglec-XII expression shares similar patterns of induction of gene expression that are encountered in polyps that are at highest risk for progression to CRCs.

**Figure 6:**
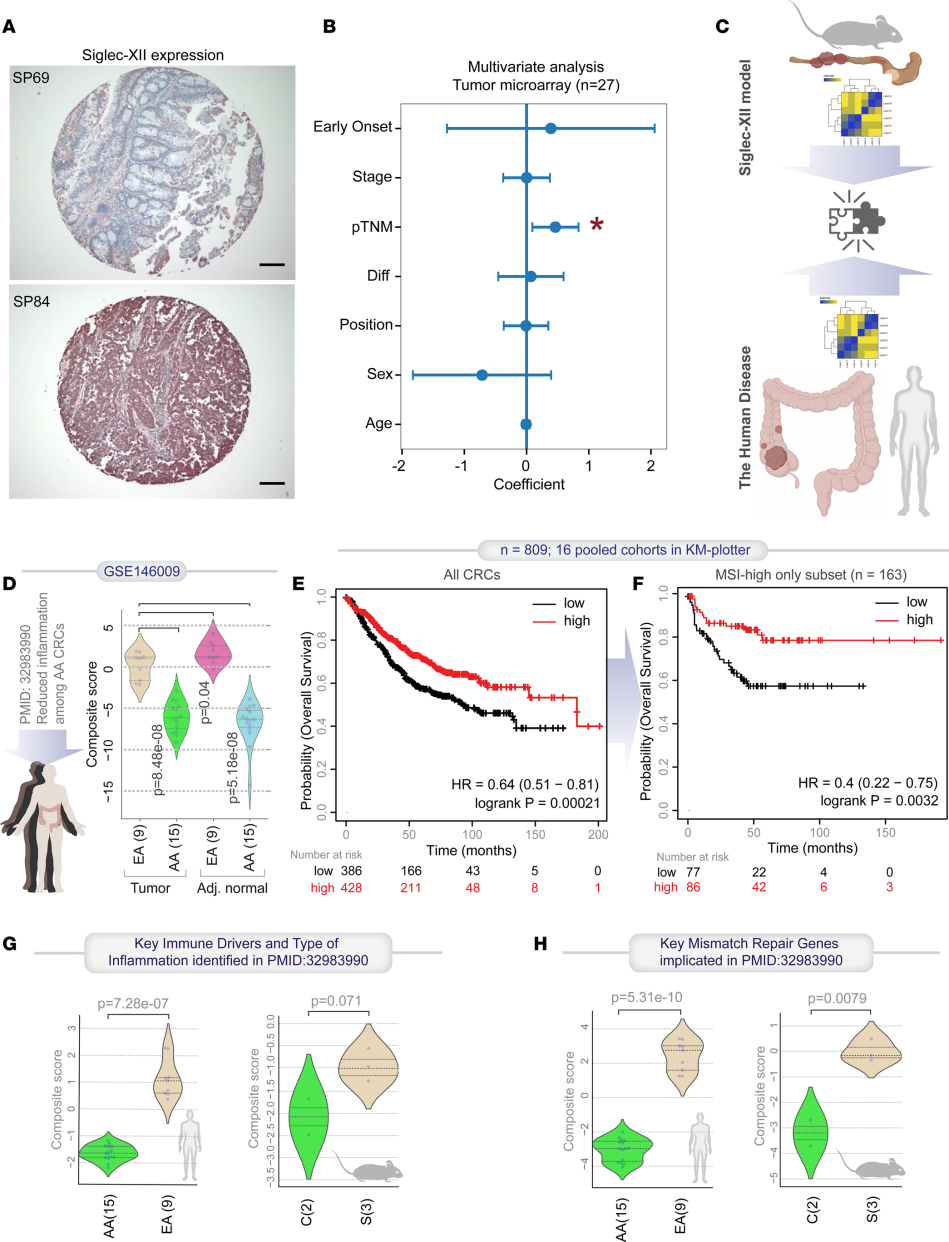
Computational Siglec-XII–positive CRCs. (**A**) Expression of Siglec-XII in human colorectal tumors was evaluated by immunohistochemistry. Representative images of tumors that were scored as negative (specimen 69) or positive (specimen 84) are shown. Scale bar: 100 μm. (**B**) Multivariate analysis of Siglec-XII positivity as a linear combination of all variables in the tumors used in this study. The coefficient of each variable (at the center) with their upper and lower bounds of 95% confidence interval (as error bars) and the *P* values from *t* tests are illustrated in the bar plot. The *P* value for each term tests the null hypothesis that the coefficient is equal to 0 (no effect). Asterisk = significant covariate. *P = 0.018. See also Supplemental Table 1 for source data. (**C**) Schematic summarizes the transcriptomics-based computational approach to find a match between model (Siglec-XII murine tumors) versus disease (human CRCs). (**D**) Violin plots display the StepMiner normalized composite scores of the DEGs (in Figure 5) in tumor and matched adjacent normal colon tissues in 15 African American (AA) and 9 European American (EA) patients. (**E** and **F**) Kaplan-Meier curves for overall and progression-free survival (Supplemental Figure 5, A and B) in patients with all CRCs (**E**) or just the MSI-high subset (**F**), stratified based on high versus low mean expression values of the DEGs in **B**. (**G** and **H**) Violin plots of the StepMiner normalized composite scores of key immune (**G**) and mismatch repair (**H**) genes that were found to be differentially expressed between the 2 ethnic groups in GSE146009 (left) and in the control (C) versus Siglec-XII (S) mouse tumors (right) (ref. 37). See also Supplemental Figure 5, C–F, for the patterns of expression of the individual genes displayed as heatmaps. Statistics: *P* values in each violin plot (**D**, **G**, and **H**) are based on 2-tailed Welch’s *t* test between comparator groups. *P* values for survival plots were determined by log-rank test.

Consistent with the fact that cancers that originate from right-sided polyps are often diagnosed at advanced stages (36), a CRC array and multivariate analyses showed that Siglec-XII positivity was significantly associated with presentation at advanced stages (pTNM; Figure 6, A and B, and Supplemental Table 1).

### Siglec-XII expression is associated with a specific ethnic subtype of CRCs.

Next, we used the 29-gene, model-derived signature as a computational tool to navigate human CRC data sets and objectively assess for a precise match in gene expression patterns in the Siglec-XII model versus human CRC subtypes (Figure 6C). We found that the 29-gene signature was significantly induced in both tumors and adjacent normal tissues from self-identified European Americans (aka “Whites” and described in the data set as “Caucasian Americans”) versus African Americans (aka “Blacks”) (Figure 6D; ROC AUC for each 1.00). This data set was first used in a study (37) that showed differential contributions of immune cells, inflammation, and mismatch repair defects among 2 ethnic groups; it is one of the studies that established what is now widely recognized as a key ethnic difference in the CRC subtypes (38). Furthermore, consistent with the fact that tumors in European Americans are more often right-sided with microsatellite instability (MSI) and carry an overall good prognosis (38), we found that high expression of the 29-gene signature was associated with a favorable outcome among all CRCs; both overall (Figure 6E) and progression-free (Supplemental Figure 5A) survival were prolonged. This favorable impact on outcome continued to hold true even when the analysis was repeated among just the MSI-high tumors (Figure 6F and Supplemental Figure 5B).

Having observed a match in the model-derived, 29-gene expression pattern in the CRC subtype to which Whites are predisposed, we asked if the converse holds true, i.e., if the key disease drivers reported in the European Americans tumors as key clinicopathological disease features are also recapitulated in our model. The study reported that European Americans, but not African Americans, develop tumors that are characterized by inflammation (high *IL1B*, *IL8*, *NFKBIE*, and *IL6ST*) and microsatellite instability (MSI-high) in the setting of altered expression of several key genes in the mismatch repair pathway (37). We found both these patterns to hold true in our mouse model (Figure 6, G and H, and Supplemental Figure 5, C–H). These findings show that the Siglec-XII model faithfully recapitulates the pathological drivers believed to be frequently seen in one ethnicity (European Americans) but not another (African Americans).

## Discussion

**Figure 7:**
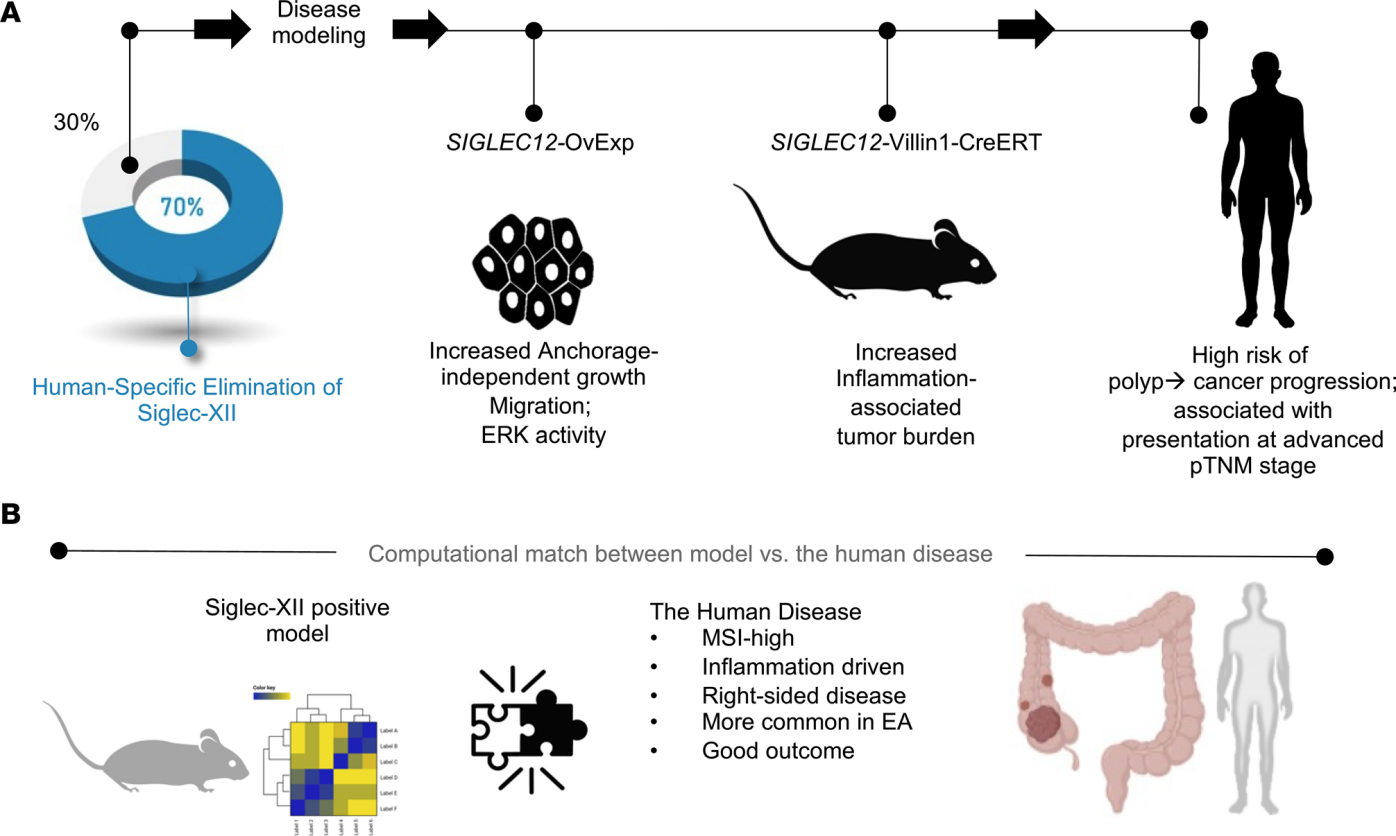
Summary of findings. (**A**) Schematic summarizes the major goal, key model systems, and key findings made using each model system in the current study. Three model systems were used, each seeking to model the oncogenic risk posed by continued Siglec-XII expression in humans (~30% of the population) despite evolutionary loss in most of the population. (**B**) Schematic summarizes the key conclusions drawn from an unbiased navigation of the human disease, performed using an objective assessment of transcriptomic data sets using a model-derived gene signature.

The discoveries we report here provide insights into the consequences of expression of the epithelial Sia binding–defective Siglec-XII in approximately 30% of the human population and how that may put them at risk of developing inflammation-driven CRCs. We show that in model systems that recapitulate most individuals who lack expression of the Siglec-XII receptor versus those who do, the expression of the receptor that is unable to bind its natural ligand has 3 key effects (see summary of findings, Figure 7): i) cancer-associated cellular phenotypes are enhanced; (ii) tumor burden is increased in mice and is associated with advanced stages of disease at diagnosis; and (iii) gene expression patterns changed in ways that mirrored with a surprising degree of precision an inflammation/environmentally driven carcinogenesis process. Because phenotypic changes in CRC-derived Caco-2 cells generally held true in prostate cancer–derived PC-3 cells, aberrant, functionally defective Siglec-XII expression in other epithelial linings may serve as a shared contributor to and/or predisposition for other inflammation-driven human carcinomas. We conclude that the persistence of Siglec-XII in humans predisposes to CRCs and likely other carcinomas, and its elimination could serve as a selection favoring survival.

It is noteworthy that besides *SIGLEC12*, there are other Siglecs that have undergone human-specific changes in functional gene status, expression, or ligand binding, which include *SIGLEC1*, *SIGLEC5/14*, *SIGLEC6*, *SIGLEC7*, *SIGLEC9*, *SIGLEC11*, *SIGLEC13*, and *SIGLEC16* (39). As with Siglec-XII, only a minority of the human population (38.7%) has a SIGLEC16 allele coding for functional protein expression, whereas the majority carries an inactive pseudogene, *SIGLEC16P*, product of a 4-nucleotide deletion disrupting the open reading frame. Although in the vast majority of these cases, we do not know how the human-specific changes impact oncogenesis, a positive association with survival in glioblastoma was found for the intact *SIGLEC16*-positive (activating Siglec) cases (9). It is possible that activating and inactivating Siglecs, when aberrantly expressed in the human population as functional or nonfunctional variants, could alter the risk of initiation and/or progression of oncogenesis in diverse organs. What we established with certainty is that in the case of Siglec-XII, its expression did not cause spontaneous carcinogenesis but predisposed to environmentally induced carcinogenesis. Its expression in established tumors, however, was associated with improved outcome.

The specific mechanism of action of the Sia binding–defective Siglec-XII in those who express it is unknown. However, taking into consideration prior reports from us (8, 18) and others (22), it is possible to highlight a relation between this cell receptor and the risk to develop carcinomas. For example, cancer-related signaling pathways were enriched in PC-3 prostate cancer cells transfected with *SIGLEC12* (18), which was accompanied by enhanced tumor growth as xenografts in nude mice (8, 18). Similar efforts to transfect the chimpanzee version of *SIGLEC12* or the arginine-restored version of human *SIGLEC12* were not successful (18), suggesting that additional components that may be critical for protein folding and targeting were also lost during evolution. The fact that Siglec-XII still recruits PTPs through phosphorylated ITIM and ITIM-like domains in its cytosolic tail (22) suggests that it retains the ability to transmit downstream signals; however, whether it does so constitutively or upon binding to hitherto-unidentified ligands remains unknown and cannot be dismissed. We show that expression of human Siglec-XII increased at least 1 type of signaling pathway in vitro in the epithelial cells (ERK/MAPK) and inflammatory cytokine signals in vivo in the murine tumors (IL-6, IL-8, IL-1β), demonstrating that despite defect in binding to its natural ligand, Siglec-XII may support some form of gain in signaling function associated with gain in pro-oncogenic phenotypes. It is possible that expression of the inhibitory Siglec-XII served as a dominant-negative receptor that sequestered tumor-suppressive SHP1/2 phosphatases (14, 15), thereby contributing to the oncogenic risk. Although we did not observe aberrant coexpression of either SHP1 or SHP2, we noted the upregulation of another member in the PTP family, i.e., *Ptpn18*; upregulation of *Ptpn18* has been reported in yet another type of CRC, i.e., early-onset CRC, and such upregulation carries worse prognosis (40).

Perhaps the most important finding of translational relevance is the degree of precision with which Siglec-XII–positive tumors (our model) recapitulated the gene expression patterns encountered in normal human colonic mucosa and in diverse human polyps and CRCs (the disease). Expression of *SIGLEC12* captured the gene expression pattern that is detected at higher levels on the right side of the colonic mucosa compared with the left. Because the 29-gene signature largely reflected mitochondrial bioenergetic processes, we suspect that this difference is due to previously demonstrated striking differences in mitochondrial bioenergetics between the right versus left colonic mucosa (41). In fact, the bioenergetic status of the right colon has been shown to mimic that seen in the normal tissue adjacent to CRCs (41). We also found that elevated expression of the 29-gene signature is encountered in polyps that carry a higher risk of progression to CRCs. It also mirrored a distinct subtype of CRCs more often encountered in European Americans; these are right-sided, primarily driven by mismatch repair defects and IL-1β/IL-8/IL-6–centric inflammation, and associated with improved outcome. Consistent with the form of disease in humans, we saw immune cell infiltrates in our mouse model. Given their active immune microenvironment and elevated expression of various checkpoint molecules, MSI-high, right-sided CRCs in Whites present as promising candidates for targeted immunotherapy with immune checkpoint inhibitors (42, 43). It is possible that either Siglec-XII or the 29-gene signature could serve as a biomarker for both prognostication and prediction of therapeutic response to immunotherapy. On the therapeutic side, Siglec-XII is a promising candidate for targeted drug delivery to cancer cells expressing it because of its limited and specific expression in only a few cell types. For example, given its ability to internalize upon antibody binding (18), coupling a toxin to the antibody presents a potentially effective strategy for advancing cancer therapy. The simple assay we developed to rapidly screen for all mutations abrogating expression using patient-derived saliva and urine samples could help identify those who may benefit (8).

Despite the insights gained, there are a few limitations of this study. The use of a handful of CRC cell lines is one; analyzing a broader range of CRC cell lines is expected to yield how this Siglec-XII phenomenon intersects with other CRC-driving genetics. Additional validation studies are also needed for dissecting the signaling pathways in the animal model; such studies are expected to establish a clearer link between Siglec-XII and its role in cancer.

Taken together, these data support the notion that Siglec-XII expression may facilitate CRC progression in humans. Similar studies need to be done with other carcinomas that also have very high incidence in humans compared with closely related apes.

## Methods

### Sex as a biological variable

In this study, we evaluated the impact of Siglec-XII expression in mice. Although only female mice were used, sex was not considered a biological variable, based on multivariate analyses of a Siglec-XII–positive human cohort (Figure 6B).

### Experimental methodologies

#### Cell lines.

Prostate (PC-3) and colorectal (Caco-2) adenocarcinoma cell lines were purchased from the American Type Culture Collection. PC-3 cells were grown in F-12K Medium (Kaighn’s Modification of Ham’s F-12 Medium) supplemented with 10% fetal bovine serum (FBS) (Gibco). Caco-2 cells were grown in Eagle Minimum Essential Medium supplemented with 20% FBS. Monolayers were routinely subcultured with Trypsin-EDTA solution (Gibco), following standard procedures. Cell cultures were maintained at 37°C in a humidified atmosphere of 5% CO_2_ and tested for contamination with Mycoplasma with the kit (Vector Laboratories). The cell lines used for the described experiments had all been maintained in tissue culture for fewer than 20 passages.

#### Establishing cell lines stably expressing Siglec-XII.

PC-3 and Caco-2 cells were transfected with PvuI linearized h*SIGLEC12*-pcDNA3.1 or empty pcDNA3.1(-) in 6-well plates using Lipofectamine 2000 (Invitrogen). At 48 hours after transfection, the cells were trypsinized and grown with 800 μg/mL G418. After 1 month in culture, expression of Siglec-XII was determined using Western blot. Cell adhesion, spontaneous spheroid formation, cell viability, cell migration, and Western blot studies were conducted using stable vector-transfected PC-3 and Caco-2 cells (h*SIGLEC12*-pcDNA3.1 or empty vector as negative control).

#### Flow cytometry.

Cell lines were collected by enzyme-free cell dissociation buffer (Thermo Fisher Scientific) and incubated with mouse anti–Siglec-XII 276, anti–Siglec-XII rabbit polyclonal antibody (AP18196PU-N, Origene), mouse IgG Isotype Control (MG1-45, BioLegend), or rabbit IgG Isotype Control (X0936, Dako) on ice for 30 minutes. Cells were washed with phosphate-buffered saline (PBS) and incubated with anti-mouse Alexa Fluor 488 (A11001, Invitrogen) or anti-rabbit Alexa Fluor 488 (A11053, Invitrogen) on ice for 30 minutes. Acquisition of data was carried out using a FACSCalibur flow cytometer (Becton Dickinson), and data were analyzed using FlowJo software.

#### SIGLEC12 frameshift mutation.

The genomic DNA was isolated from cell lines. The frameshift deletion mutation of *SIGLEC12* was analyzed using PCR. The primers used to amplify the *SIGLEC12* locus were 5′ACCCCTGCTCTGTGGGAGAGT3′ (forward) and 5′AGGATCAGGAGGGGCATCCAAGGTGC3′ (reverse). The PCR was performed using the Phusion High-Fidelity Polymerase kit (Thermo Fisher Scientific). The amplified product was purified using the QIAquick PCR Purification Kit (QIAGEN) and sent for sequencing to EtonBio. The sequencing was performed using the primer 5′CTCTCTCTGGTGTCTCTGATGC3′ (reverse).

#### Cell adhesion assay.

Cell adhesion was measured using crystal violet staining. Cells were harvested with an enzyme-free cell dissociation buffer and seeded at a concentration of 4 × 10^4^ cells/well in complete medium in a 96-well plate. After incubation at 37°C at 0.5, 1, and 1.5 hours, the cells were washed with PBS, and nonadherent cells were removed by aspiration. Adherent cells were stained with a 0.5% (w/v) crystal violet solution with 20% (v/v) methanol. After washes, the dye was solubilized with 10% (v/v) methanol and 5% (v/v) acetic acid, and the absorbance was measured at 595 nm by EnSpire Multimode Plate Reader (PerkinElmer).

#### Spontaneous spheroid formation.

Cells were harvested and passed through a 40 μM cell strainer (Corning). Cells were plated at a density of 3,000 cells in 100 μL of growth media per well using 96-well spheroid microplates. Spheroid cultures were photographed, and cell viability was measured at day 4, 8, and 10. The same seeding methods were used for all cell lines.

#### Cell viability assay.

The CellTiter-Glo 3D Cell Viability Assay protocol was followed (Promega). The CellTiter-Glo 3D Cell Viability Assay is a homogeneous method to determine the number of viable cells in 3D cell culture based on quantitation of the ATP present, which is a marker for the presence of metabolically active cells. Briefly, spheroids were transferred to white plates, and CellTiter-Glo 3D reagent was added directly into wells in a 1:1 dilution. The solutions were well mixed by shaking the plate for 5 minutes then incubated at room temperature for 25 minutes. After incubation, the generated luminescent signal was read and analyzed using the EnSpire Multimode Plate Reader.

#### Cell migration assay.

After overnight starvation, 1 × 10^4^ PC-3 or 2 × 10^4^ Caco-2 cells previously transfected with h*SIGLEC12*-pcDNA3.1or empty pcDNA3.1(-) were seeded into the Transwell inserts (HTS Transwell-96 Permeable Support with 8.0 μm Pore Polyester Membrane, Corning) in serum-free medium. The lower chamber was filled with 10% FBS–containing medium. Stationary cells were removed from the upper surface of the membranes with a cotton swab. Cells that migrated to the lower surface were fixed and stained with crystal violet. Migrating cells were counted in 5 randomly selected fields and normalized to control.

#### Western blotting and antibodies.

Cells were homogenized on ice in RIPA lysis buffer (Cell Signaling Technology, CST) supplemented with protease and phosphatase inhibitors (CST). Protein concentration was quantified using a Pierce bicinchoninic acid protein assay kit (Thermo Fisher Scientific). Equal amounts of proteins were resolved by SDS-PAGE and transferred onto PVDF membranes (Bio-Rad). The membranes were blocked in TBS with 0.1% Tween 20 (TBST, CST) and 0.5% BSA (MilliporeSigma) for 1 hour at room temperature and then incubated with the primary antibodies at 4°C overnight. The primary antibodies used for immunoblotting were anti–β-Actin (4970, 1:10,000, CST), anti–phospho-p44/42 MAPK (Erk1/2) (Thr202/Tyr204) antibody (9101, 1:1,000, CST), anti-p44/42 MAPK (Erk1/2) antibody (9102, 1:1,000, CST), and anti–Siglec-XII (AP18196PU-N, 1:2,000, Origene). Then, membranes were incubated with IRDye 800CW Goat anti-Rabbit IgG secondary antibody (926-32211, 1:15,000, LI-COR Biosciences). Protein bands were visualized using Odyssey Imager (LI-COR Biosciences).

#### Mouse strains.

Villin1-Cre–transgenic mice, with a Cre recombinase gene introduced under the promoter of the Villin1 gene (24), were acquired from The Jackson Laboratory. Human *SIGLEC12* conditional knockin was produced by Cyagen. The guide RNA to mouse ROSA26 gene, the donor vector containing BGH pA-Kozak-human *SIGLEC12*CDSloxP-SV40 early pA-EGFP-*loxP*-CAG promoter cassette, and Cas9 mRNA were coinjected into fertilized mouse eggs to generate targeted conditional knockin offspring. F_0_ founder animals were identified by PCR followed by sequence analysis, which were bred to wild-type mice to test germline transmission and F_1_ animal generation. The *SIGLEC12*-knockin mice presents the *egfp* gene (including stop codon) flanked between 2 *loxP* sites, and it is upstream of the Siglec-XII coding sequence. Mice with 1 floxed allele for *SIGLEC12* were crossed with Villin1-Cre, to generate Villin1-Cre with heterozygous floxed *SIGLEC12* progeny. The littermates containing only Villin1-Cre were used as controls.

#### Tamoxifen preparation and administration.

Tamoxifen (MilliporeSigma) was prepared as described previously (44). Eight-week-old mice were i.p. injected with 100 μL of tamoxifen stock solution (10 mg/mL) for 5 consecutive days and sacrificed either after 12 or 87 days of the first injection.

#### Immunohistochemistry studies.

Slides with multitissue arrays of different human carcinomas were obtained from Super Bio Chips. The sections were deparaffinized and blocked for endogenous biotin and peroxidase. Citrate buffer pH 6.0 was used for heat-induced epitope retrieval. A 5-step signal amplification method was used, consisting of the application of mouse monoclonal anti–Siglec-XII antibody (clone 276, described earlier) (18), followed by biotinylated donkey anti-mouse, HRP (Jackson ImmunoResearch Laboratories, catalog 715-065-150), streptavidin, followed by application of the enzyme biotinyl tyramide, and then, labeled streptavidin (Jackson ImmunoResearch Laboratories).

For mouse samples, tissues were frozen in OCT and processed for frozen sections using the Leica cryostat. Slides were fixed for 1 minute in acetone and, after 3 washes with TBST, incubated with anti–Siglec-XII (AP18196PU-N, Origene) antibody for 30 minutes at room temperature. After 3 washes with TBST, slides were incubated with Peroxidase AffiniPure Goat Anti-Rabbit IgG (111-035-045,Jackson ImmunoResearch Laboratories) for 30 minutes at room temperature. For both human tissue array and frozen mouse tissue sections, the AEC kit (Vector Laboratories) was used as substrate, nuclear counterstain was carried out with Mayer’s hematoxylin, and the slides were aqueous mounted for digital photographs, taken using the Olympus BH2 microscope.

#### Modeling colorectal carcinogenesis.

Eight-week-old female mice received 5 days of treatment (10 mg/mL) with tamoxifen followed by an i.p. injection (10 mg/kg body weight) of AOM (MilliporeSigma) followed by 5 days of DSS (MP Biomedicals) treatment (2.0%) and 14 days of recovery, as described previously (28). This cycle was repeated 3 times. After the fourth DSS cycle (87 days), mice were sacrificed, and organs were harvested for various analyses. This included small intestines, colons, kidneys, livers, lungs, and spleens. The intestines were opened and examined for the presence of tumors, and the number of intestinal tumors was assessed. The size of the tumors was determined by ImageJ software (NIH).

#### Tissue histology.

Colon, kidney, liver, spleen, and lung samples were immediately fixed in 10% neutral buffered formalin and processed into paraffin blocks and sectioned at 3 μm using a microtome and placed on slides. These slides were used for H&E staining. Digital photographs of H&E were taken using the Keyence BZ-9000E microscope. The Keyence microscope system was used to capture digital images at low power, and the images were merged to obtain the final image of the roll of mouse intestine.

### Computational methodologies

#### Curation of publicly available data sets.

Several publicly available microarrays and RNA-Seq databases were downloaded from the NCBI GEO server. Gene expression summarization was performed by normalizing Affymetrix platforms by robust multichip average and RNA-Seq platforms by computing transcripts per million (TPM) values whenever normalized data were not available in GEO. We used log_2_(TPM + 1) as the final gene expression value for analyses. GEO accession numbers are reported in figures and text. For the data set (GSE146009; 15 African American and 18 Caucasian American samples) containing RNA-Seq data generated by TruSeq Stranded mRNA Library Prep Kit, we obtained it from NCBI GEO and subsequently cleaned to exclude paired tumor and normal mucosa samples with the mapping rate greater than 90% (all 15 African American and 9 Caucasian American samples passed the quality control check). Caucasians were defined as Americans with European ancestry; African Americans were defined as having any amount of ancestry contribution from Africa.

#### RNA-Seq on cells and mouse colons.

Caco-2 cells and mouse tissues (distal colon after tamoxifen administration for baseline, and mouse colon tumors from AOM/DSS carcinogenesis protocol) were subjected to mRNA extraction using RNeasy Plus Mini Kit (QIAGEN). Sample quality was evaluated by the TapeStation system (Agilent). Transcriptomic analysis was performed on RNA libraries prepared from samples not expressing or expressing Siglec-XII using the Illumina Stranded mRNA Prep. Each sample was used to prepare 3 separate technical replicate libraries for sequencing. Libraries were sequenced at 2 × 100 bp on NovaSeq 6000 (Illumina). Reads were mapped to human reference genome Hg19 using kallisto 0.44.0 pipeline. Mapped reads were counted at the gene level using featureCounts v1.5.220, and counts were analyzed using DESeq2 v1.14.1.21. Sample clustering was confirmed by principal component analysis, which is an unsupervised learning algorithm technique used to examine the interrelations among a set of variables. DEGs with a *P* ≤ 0.05 and fold-change ≥ 2 were then selected for further examination.

#### Gene expression analyses.

The expression levels of all genes in these data sets were converted to binary values (high or low) using the StepMiner algorithm (45), which undergoes an adaptive regression scheme to verify the best possible up and down steps based on sum-of-square errors. The steps are placed between data points at the sharpest change between expression levels, which gives us the information about threshold of the gene expression-switching event. To fit a step function, the algorithm evaluates all possible steps for each position and computes the average of the values on both sides of a step for the constant segments. An adaptive regression scheme is used that chooses the step positions that minimize the square error with the fitted data. Finally, a regression test statistic is computed as follows:


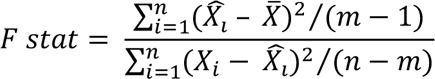


Where *X_i_* for *i* = 1 to *n* are the values, for *i* = 1 to *n* are fitted values. M is the degrees of freedom used for the adaptive regression analysis. is the average of all the values:


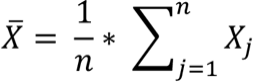


For a step position at *k*, the fitted values are computed by using:


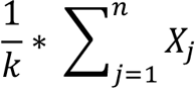


for *i* = 1 to *k* and


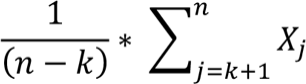


for *i* = *k* + 1 to *n*.

Gene expression values were normalized according to a modified *Z*-score approach centered around StepMiner threshold: formula = (expr – SThr)/(3 × stddev). The normalized expression values for all genes were added together to create the final score for the gene signature. The samples were ordered based on the final signature score. DEGs were identified using DESeq2 package in R. Standard *t* tests were performed using python scipy.stats.ttest_ind package (version 0.19.0) with Welch’s 2-sample *t* test (unpaired, unequal variance [equal_var = False] and unequal sample size) parameters. Multiple-hypothesis correction was performed by adjusting *P* values with statsmodels.stats.multitest.multipletests (fdr_bh: Benjamini-Hochberg principles). Pathway analysis of gene lists was carried out via the Reactome database and GO Biological Processes.

### Unsupervised clustering and heatmaps

Expression patterns of the genes that are differentially expressed in African American and Caucasian American samples (in GSE146009) and *SIGLEC12*-expressing and control groups, before or after AOM/DSS challenge, are clustered without bias based on their *z*-normalized counts per million expression values, in all the samples. The data are visualized using the seaborn clustermap package (v 0.12) in python.

### Multivariate analyses

To assess which demographic and clinicopathologic factor(s) may influence Siglec-XII expression in CRCs, multivariate regression has been performed on a tumor microarray data set. Multivariate analysis models the *SIGLEC12* expression in samples (base variable) as a linear combination of all other metadata that was associated with each tumor, i.e., clinical (stage, pTNM, location), demographic (age/gender), or histopathological parameters. Here, the stats models module from python has been used to perform ordinary least-squares regression analysis of each of the variables. The *P* value for each term tests the null hypothesis that the coefficient is equal to 0 (no effect).

### Kaplan-Meier survival plots

Survival analysis was performed using “Use multiple genes” options on Kaplan-Meier Plotter (46) and running the analysis on the *SIGLEC12* gene signature using the default setting using the mean expression of the genes.

### Statistics

Statistical significance was calculated using Prism 10 statistical software (GraphPad). The data presented in this study are expressed as mean values ± SD. Normality test was performed prior to statistical test. For comparisons between 2 independent samples, 2-tailed *t* test was used. For multiple comparisons, 2-way ANOVA, followed by Tukey’s multiple comparisons test, was performed. The data correspond to at least 3 independent experiments. A statistically significant value was defined as *P* < 0.05.

### Study approval

Mice were housed at an animal facility of the University of California, San Diego (UCSD). All mouse procedures were approved by the Institutional Animal Care and Use Committee.

### Data availability

RNA-Seq data have been made available publicly through the NCBI GEO repository (GSE262088) and in the Supporting Data Values XLS file.

## Author contributions

HAC, NV, AV, and PG conceptualized the project. HAC performed the experiments and analyzed the results. SS and PG conducted all computational analyses in this work. HAC, NV, AV, SS, and PG prepared display items for data visualization. HAC, NV, AV, and PG wrote the original draft of the manuscript. HAC, NV, AV, SS, ALV, and PG provided input and edited and revised the manuscript. HAC, NV, AV, SS, ALV, and PG approved the final version of the manuscript. AV and PG coordinated and supervised all parts of the project.

## Supplementary Material

Supplemental data

Unedited blot and gel images

Supporting data values

## Version 1 07/11/2024

In-Press Preview

## Version 2 08/22/2024

Electronic publication
