# Supplementary material for: Human-specific elimination of epithelial Siglec-XII suppresses the risk of inflammation-driven colorectal cancers
Source: JCI Insight. 2024 Jul 11;9(16):e181539. doi: 10.1172/jci.insight.181539 (PMC11343606; doi:10.1172/jci.insight.181539)
Supplement: Supplemental data [file jciinsight-9-181539-s057.pdf]

SUPPLEMENTARY ONLINE MATERIALS

Human-Specific Elimination of Epithelial Siglec-XII Suppresses the Risk of Inflammation Driven Colorectal Cancers

AUTHORS: Cuello Hector A[1, 2], Sinha Saptarshi[1, 3, 4], Verhagen Andrea L[1, 2], Varki Nissi[2, 5], Varki Ajit[1-3, 6*], Ghosh Pradipta[1, 3, 4, 7*].

AFFILIATIONS:
[1]Department of Cellular and Molecular Medicine, University of California San Diego, CA, USA;
[2]Glycobiology Research and Training Center, University of California San Diego, CA, USA;
[3]Department of Medicine, University of California San Diego, CA, USA;
[4]Moore's Comprehensive Cancer Center, University of California San Diego, CA, USA;
[5]Department of Pathology, University of California San Diego, CA, USA;
[6]Center for Academic Research and Training in Anthropogeny, University of California San Diego, CA, USA;
[7]HUMANOID Center of Research Excellence (CoRE), University of California San Diego, CA, USA;

Short Title: Siglec-XII expression drives inflammation-associated CRCs.

* **CORRESPONDING AUTHOR CONTACT INFORMATION**

Pradipta Ghosh, M.D.; Professor, Departments of Medicine and Cell and Molecular Medicine, University of California San Diego; 9500 Gilman Drive (MC 0651), George E. Palade Bldg, Rm 232; La Jolla, CA 92093. **Phone**: 858-822-7633. **Email:** prghosh@ucsd.edu

Ajit Varki; Professor, Department of Cell and Molecular Medicine, University of California San Diego; 9500 Gilman Drive, Biomedical Research Facility II; La Jolla, CA 92093. **Phone**: 858-534-2214. **Email:** a1varki@health.ucsd.edu.

STATEMENT ON CONFLICT OF INTERESTS
The authors declare no competing interest with the content of this manuscript.

CATALOG OF MATERIALS:

Supplementary tables: 1
Supplementary Figures and Legends: 5

SUPPLEMENTAL

Microarray Number	Age	Sex	Location	Differentiation	pT	pN	pM	pTNM	Stage	SiglecXII	Early onset Yes 1; No 0
69	44	F	5	AdenoCA well diff	T1	N0	M0	T1N0M0	I	0	1
70	52	M	1	AdenoCA well diff	T3	N0	M0	T3N0M0	II A	0	0
71	71	F	5	AdenoCA well diff	T3	N0	M0	T3N0M0	II A	2	0
72	82	F	2	AdenoCA well diff	T3	N2	M0	T3N2M0	III C	2	0
73	50	F	5	AdenoCA well diff	T1	N0	M0	T1N0M0	II A	0	0
74	48	F	1	AdenoCA well diff	T3	N1	M0	T3N1M0	II B	0	0
75	54	F	7	AdenoCA well diff	T3	N1	M1	T3N1M1	IV	0	0
76	73	F	7	AdenoCA well diff	Unk	Unk	Unk	Unk	Unk	1	0
77	62	F	3	AdenoCA mod diff	T3	N0	M0	T3N0M0	II A	2	0
78	46	M	5	AdenoCA mod diff	T3	N0	M0	T3N0M0	II A	0	0
79	73	F	2	AdenoCA mod diff	T3	N0	M0	T3N0M0	II A	2	0
80	72	F	2	AdenoCA mod diff	T3	N1	M0	T3N1M0	III B	0	0
81	37	F	1	AdenoCA poorly diff	T3	N1	M0	T3N1M0	III B	2	1
82	44	M	1	AdenoCA well diff	T3	N2	M0	T3N2M0	III C	1	1
83	69	F	6	AdenoCA well diff	T3	N0	M0	T3N0M0	II A	3	0
84	31	M	2	AdenoCA poorly diff	T3	N0	M0	T3N0M0	II A	3	1
85	42	F	7	AdenoCA well diff	Unk	Unk	M1	M1	IV	2	1
86	50	M	1	AdenoCA mod diff	Unk	Unk	M1	M1	IV	0	0
87	51	F	4	AdenoCA well diff	T3	N2	M0	T3N2M0	III C	0	0
88	75	F	4	AdenoCA well diff	T2	N0	M0	T2N0M0	I	0	0
89	43	M	4	AdenoCA well diff	T3	N1	M0	T3N1M0	III B	1	1
90	60	M	4	AdenoCA well diff	T3	N1	M0	T3N1M0	III B	0	0
91	55	F	4	AdenoCA well diff	T3	N1	M0	T3N1M0	III B	2	0
92	63	F	4	AdenoCA mod diff	T3	N2	M0	T3N2M0	III C	0	0
93	55	M	4	AdenoCA mod diff	T3	N0	M0	T3N0M0	II A	2	0
94	63	F	4	AdenoCA mod diff	T3	N0	M0	T3N0M0	II A	2	0
96	70	M	4	AdenoCA mod diff	T3	N0	M0	T3N0M0	II A	0	0

	LOCATION (KEY)
**1**	Ascending
**2**	Cecum
**3**	Descending
**4**	Rectum
**5**	Sigmoid
**6**	Transverse
**7**	Colon (undefined)

Supplemental Table 1. Information of CRC specimens present in tissue microarray. T= Tumor, N= Node, M=Metastasis.

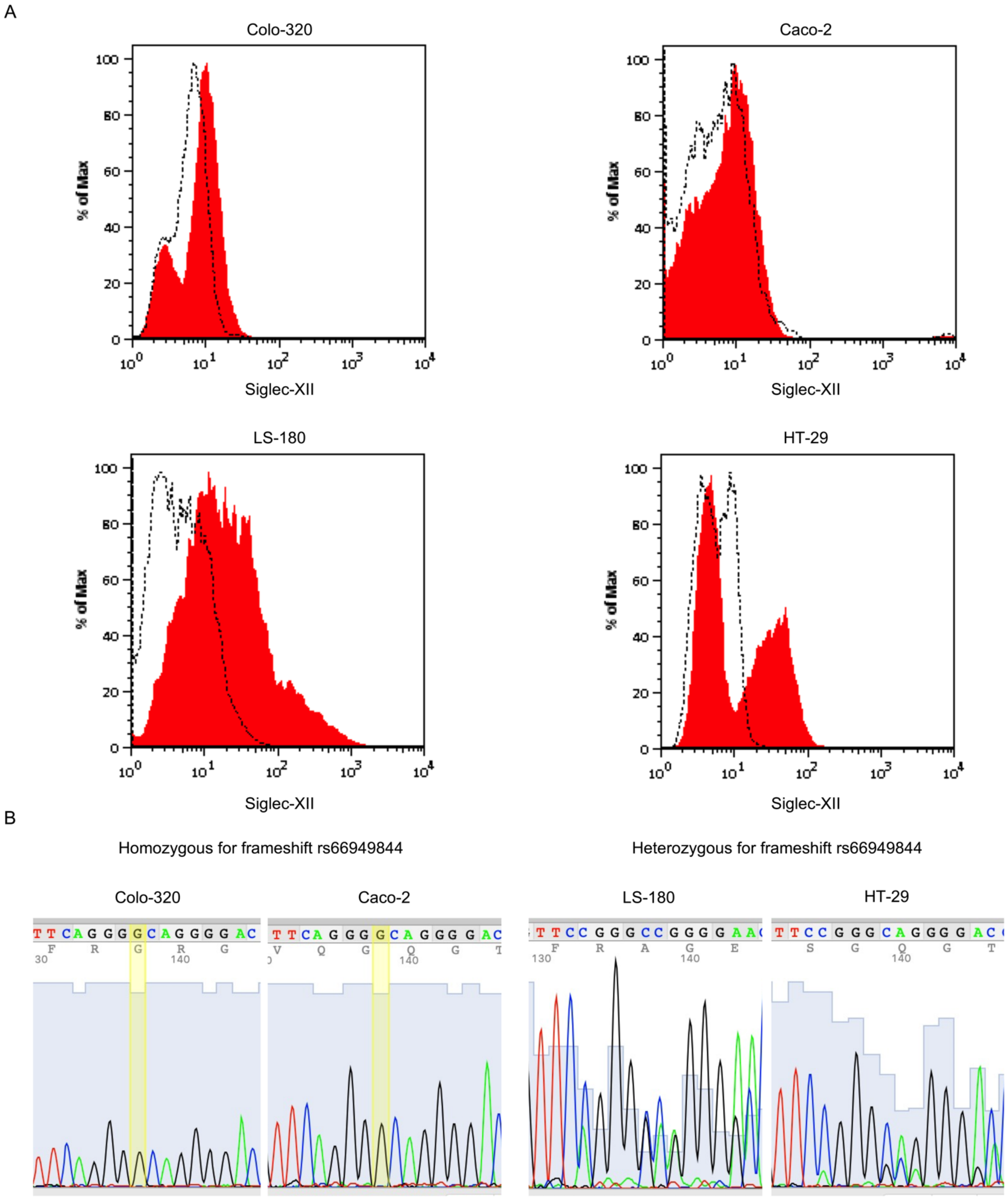

Supplemental Figure S1. Analysis of Siglec-XII expression in human colorectal cancer cell lines. **A.** Representative FACS plot of Siglec-XII expression in Colo 320, Caco-2, LS-180 and HT-29 cells. **B**. Sequencing Chromatograms of Colo 320, Caco-2, LS-180 and HT-29 cells showing analysis of frameshift mutation that causes loss of *SIGLEC12* expression.

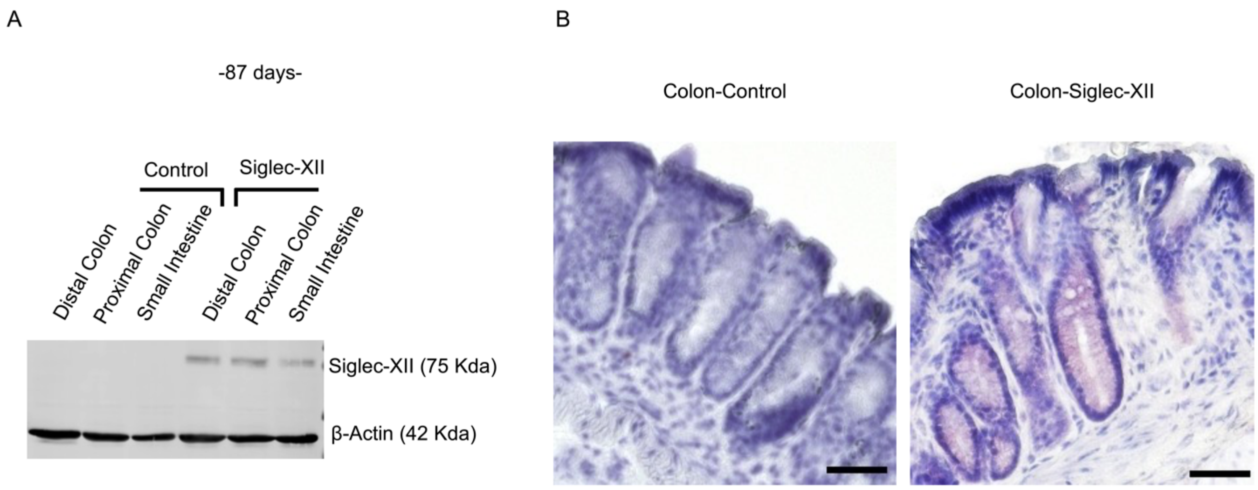

Supplemental Figure S2. Creation and validation of a transgenic knock-in *SIGLEC12* murine model that expresses Siglec-XII in the small and large intestine. **A.** Western blot for Siglec-XII and β-Actin on transgenic mouse and control tissues at day 87 post induction using Tamoxifen. **B.** Expression of Siglec-XII in mouse tissue evaluated by immunohistochemistry at day 87 post induction using Tamoxifen. Scale bar: 50 μm.

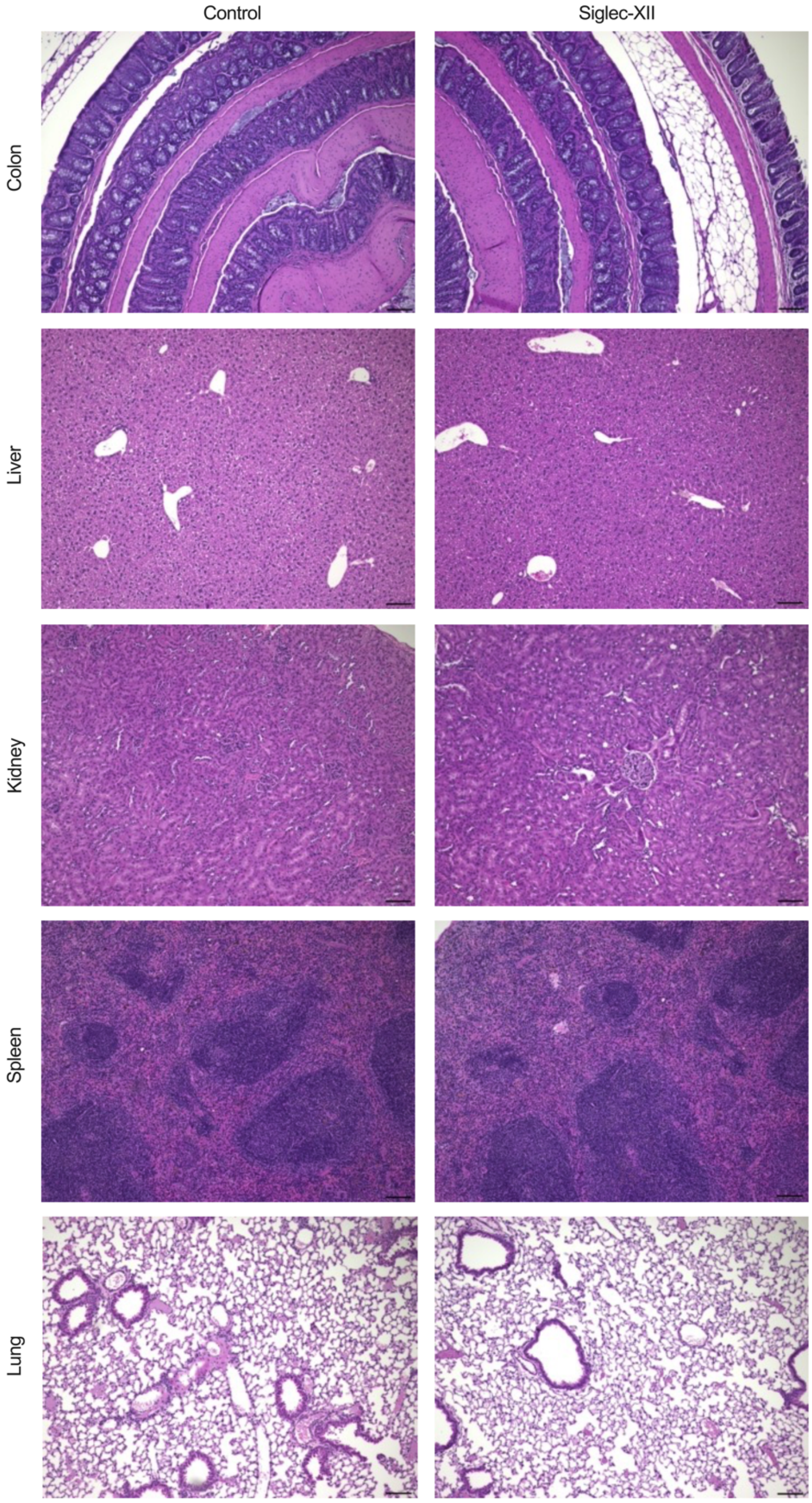

Supplemental Figure S3. The conditional expression of Siglec-XII caused no histological changes in transgenic mice or in controls. H&E of colon, liver, kidney, spleen and lung of control and Siglec-XII transgenic mice at day 87 post induction using Tamoxifen. Scale bar: 100 µm.

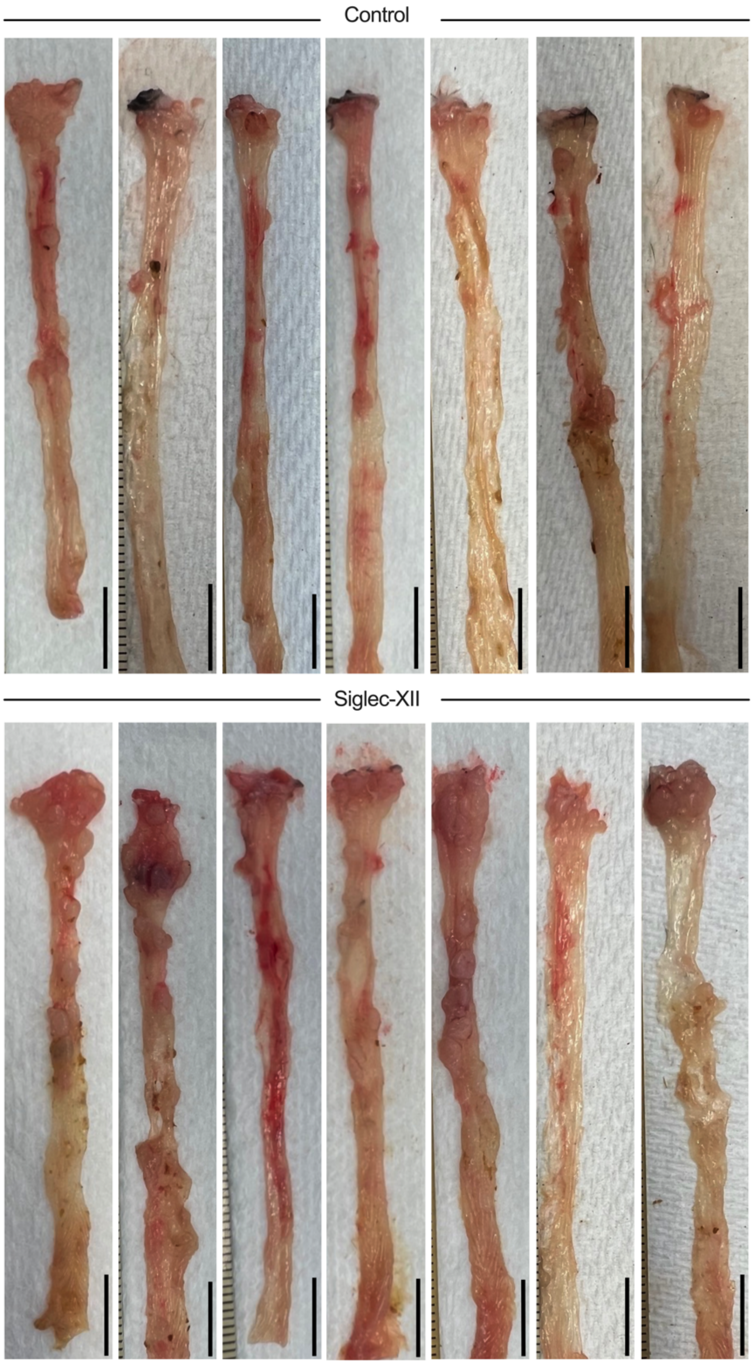

Supplemental Figure S4. Transgenic knock-in *SIGLEC12* mice are at greater risk of inflammation-associated colorectal cancers. Pictures of colonic tissue from control and Siglec-XII-expressing mice subjected to tamoxifen administration and carcinogenesis protocol (AOM/DSS). Scale bar: 1 cm.

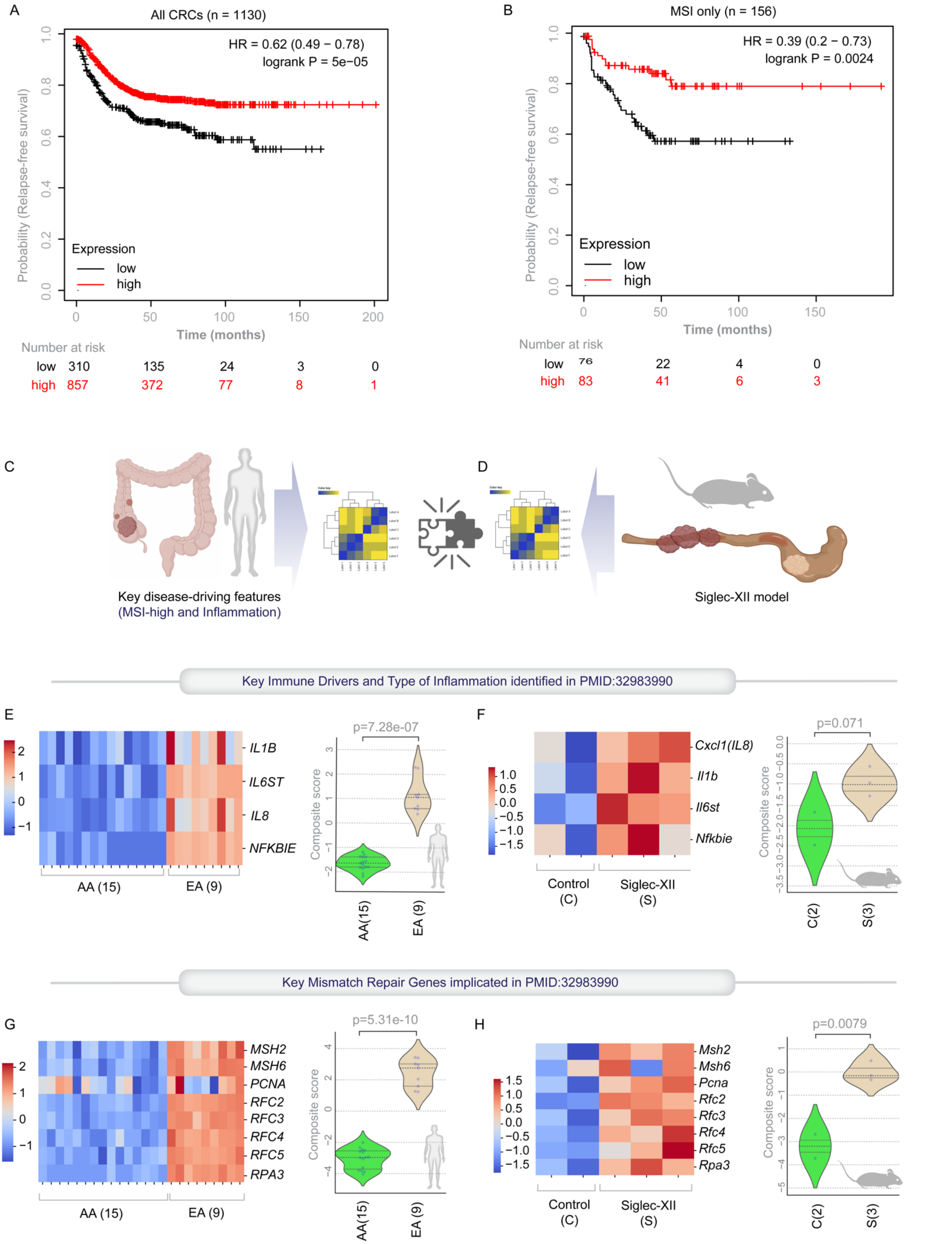

Supplemental Figure S5. A-B. Kaplan-Meier curves for progression-free survival in patients with all CRCs (A) or just the MSI-high subset (B), stratified based on high vs low mean expression values of the DEGs in **4B**. **C-D**. Schematic summarizes the transcriptomics-based computational approach to find a match between disease (human CRCs; C) vs. model (Siglec-XII murine tumors; D). **E-H.** Heatmaps of z-score normalized expression patterns (left) and Violin plots of the composite scores (right) of key immune (E-F) and mismatch repair (G-H) genes that were found to be differentially expressed between the two ethnic groups in GSE146009 (E, G) and in the control vs Siglec-XII mouse tumors (F, H). The Violin plots are same as those shown in **Figure 5G-H**.

Statistics: *p* values for survival plots were determined by log rank test. *p* values in each violin plot (E-H) are based on Welch's T-test between comparator groups.
