# Supplementary material for: Human-specific elimination of epithelial Siglec-XII suppresses the risk of inflammation-driven colorectal cancers
Source: JCI Insight. 2024 Jul 11;9(16):e181539. doi: 10.1172/jci.insight.181539 (PMC11343606; doi:10.1172/jci.insight.181539)

Full unedited gel for Figure 2H

PC-3 1= pcDNA3.1(-); 2= Siglec-XII

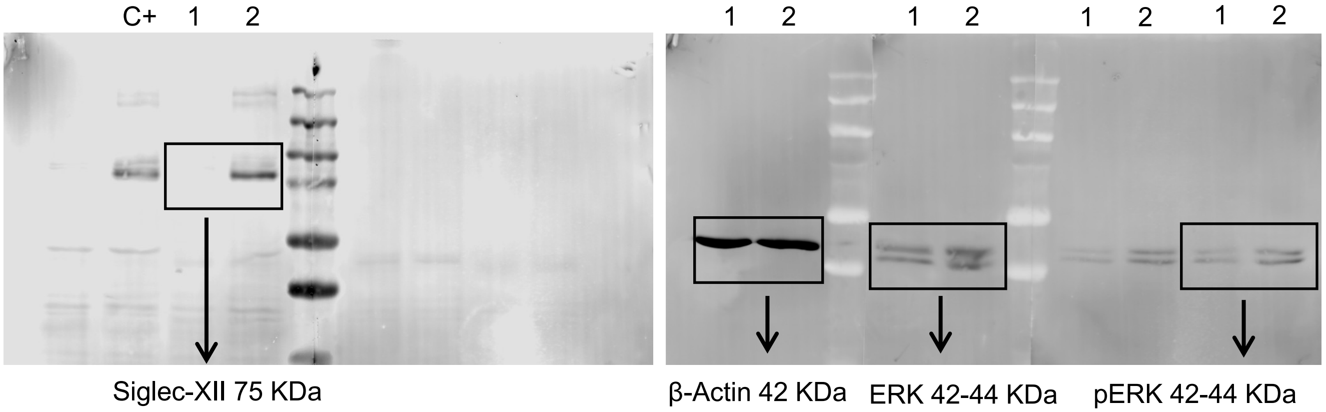

Full unedited gel for Figure 2J

Caco-2 1= pcDNA3.1(-); 2= Siglec-XII

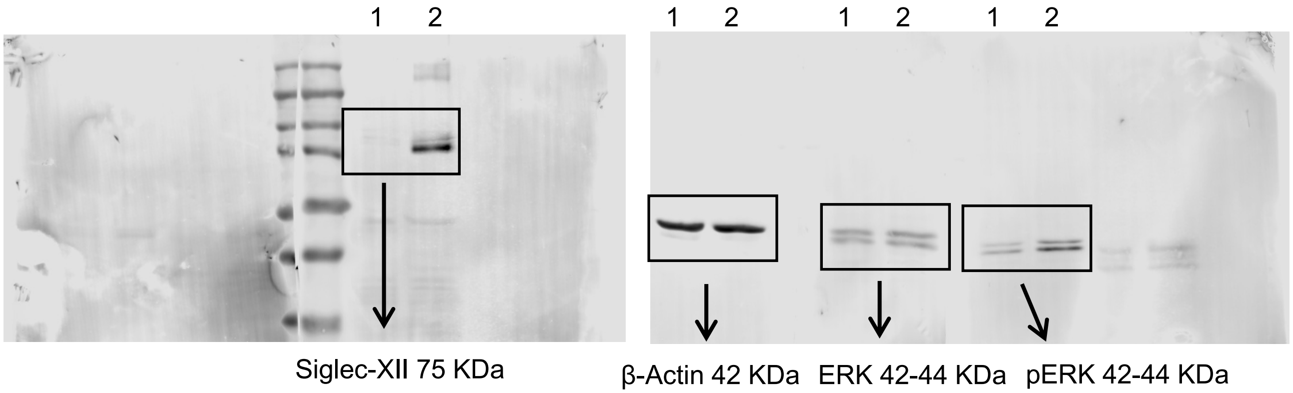

Full unedited gel for Figure 3C

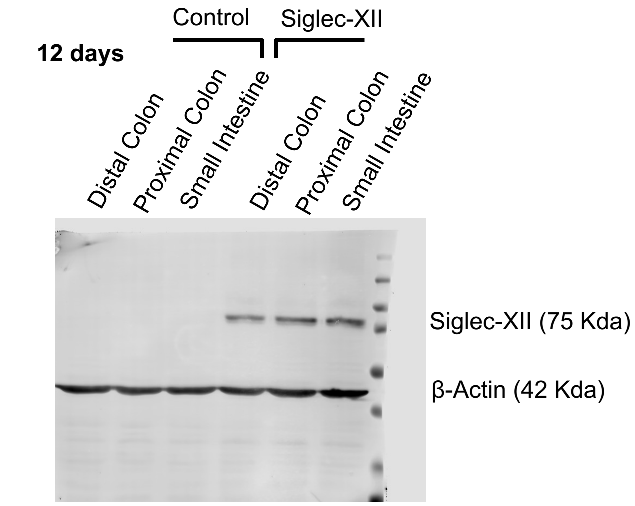

Full unedited gel for Figure S2A

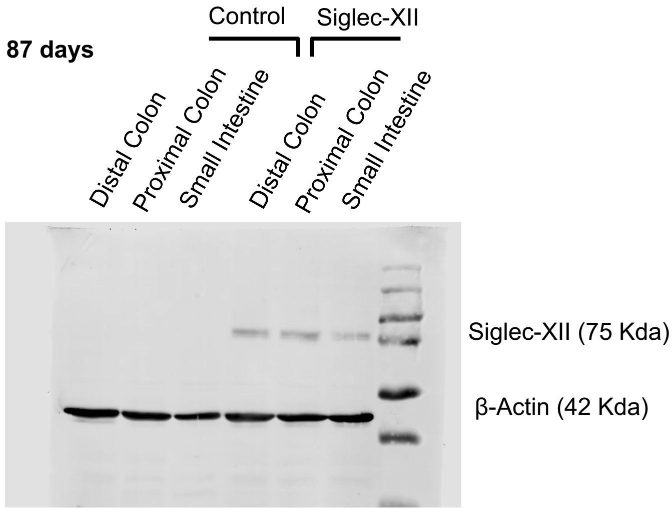

Supplement: Unedited blot and gel images [file jciinsight-9-181539-s058.pdf]
